# Exercise promotes brain health: a systematic review of fNIRS studies

**DOI:** 10.3389/fpsyg.2024.1327822

**Published:** 2024-04-10

**Authors:** Qi-Qi Shen, Jin-Mei Hou, Tong Xia, Jing-Yi Zhang, Dong-Ling Wang, Yuan Yang, Rui Luo, Zhen-Lei Xin, Heng-chan Yin, Lei Cui

**Affiliations:** College of P. E. and Sports, Beijing Normal University, Beijing, China

**Keywords:** exercise, physical activity, brain plasticity, functional near-infrared spectroscopy (fNIRS), review

## Abstract

Exercise can induce brain plasticity. Functional near-infrared spectroscopy (fNIRS) is a functional neuroimaging technique that exploits cerebral hemodynamics and has been widely used in the field of sports psychology to reveal the neural mechanisms underlying the effects of exercise. However, most existing fNIRS studies are cross-sectional and do not include exercise interventions. In addition, attributed to differences in experimental designs, the causal relationship between exercise and brain functions remains elusive. Hence, this systematic review aimed to determine the effects of exercise interventions on alterations in brain functional activity in healthy individuals using fNIRS and to determine the applicability of fNIRS in the research design of the effects of various exercise interventions on brain function. Scopus, Web of Science, PubMed, CNKI, Wanfang, and Weipu databases were searched for studies published up to June 15, 2021. This study was performed in accordance with the PRISMA guidelines. Two investigators independently selected articles and extracted relevant information. Disagreements were resolved by discussion with another author. Quality was assessed using the Cochrane risk-of-bias method. Data were pooled using random-effects models. A total of 29 studies were included in the analysis. Our results indicated that exercise interventions alter oxygenated hemoglobin levels in the prefrontal cortex and motor cortex, which are associated with improvements in higher cognitive functions (e.g., inhibitory control and working memory). The frontal cortex and motor cortex may be key regions for exercise-induced promotion of brain health. Future research is warranted on fluctuations in cerebral blood flow during exercise to elucidate the neural mechanism underlying the effects of exercise. Moreover, given that fNIRS is insensitive to motion, this technique is ideally suited for research during exercise interventions. Important factors include the study design, fNIRS device parameters, and exercise protocol. The examination of cerebral blood flow during exercise intervention is a future research direction that has the potential to identify cortical hemodynamic changes and elucidate the relationship between exercise and cognition. Future studies can combine multiple study designs to measure blood flow prior to and after exercise and during exercise in a more in-depth and comprehensive manner.

## 1 Introduction

Exercise intervention is a convenient and adaptive approach to effectively enhance the cognitive function and emotion of individuals (Verburgh et al., 2014; Kawagoe et al., 2017). Indeed, an increasing number of studies have demonstrated its beneficial effects on the healthy development of brain function (Mandolesi et al., 2018; Chen, 2020). Recent studies have predominantly focused on the variations in cognitive function and brain functional activity, such as cerebral blood flow, before and after exercise intervention (Fujihara et al., 2021; Kim et al., 2021; Zhang et al., 2021). Exploring real-time alterations in cerebral blood flow during exercise interventions can reveal hemodynamic changes (Endo et al., 2013; Eggenberger et al., 2016; Carius et al., 2020) and execution (Chen et al., 2017; Coetsee and Terblanche, 2017; Yang et al., 2020) and enhance our understanding of the mechanism underlying the effects of exercise on the brain.

The development of functional near-infrared spectroscopy (fNIRS) has enabled the exploration of hemodynamic changes in cerebral blood flow during exercise interventions. Specifically, it allows non-invasive monitoring of brain tissue oxygenation and hemodynamics (Hoshi, 2005) and possesses distinct advantages over other neuroimaging modalities, such as electroencephalography (EEG) and functional magnetic resonance imaging (fMRI). In addition, it balances both temporal resolution and spatial resolution and is comparatively less sensitive to motion (Leff et al., 2011; Scarapicchia et al., 2017). Previous exercise intervention studies using fNIRS devices largely focused on exercise interventions such as walking (Hamacher et al., 2015), posture, and walking (Herold et al., 2017), which are practical within the laboratory setting. Given the diversity in experimental designs, the effects of exercise on the brain exhibit substantial variability.

The application of fNIRS in the field of sport and exercise psychology is heterogeneous due to variations in the utilization of fNIRS and experimental design. Therefore, to improve uniformity across different studies investigating the influence of exercise on brain functional activity, this review aimed to examine studies that employed near-infrared spectroscopy to detect changes in brain hemodynamics before, during, and after exercise. The purpose of this review was as follows: (1) offer recommendations regarding study designs and research related to fNIRS technology in exercise intervention studies; (2) analyze the designs of various exercise protocols and compare the results obtained after or during exercise; and (3) evaluate the characteristics of changes in cerebral blood flow after and during exercise. Overall, the objective of this review was to investigate the effects of various exercise interventions on alterations in brain functional activity from different perspectives (before and after exercise vs. during exercise).

## 2 Methods

This systematic review was performed and reported in accordance with the Preferred Reporting Items for Systematic Reviews and Meta-Analysis (PRISMA) guidelines (Page et al., 2021) and the Cochrane Collaboration Handbook (Higgins et al., 2019).

### 2.1 Search strategy

Two reviewers (J.M.H. and T.X.) conducted an independent literature search to screen related studies. The third reviewer, Q.Q.S., resolved disagreements by arbitration.

Scopus, Web of Science, PubMed, CNKI, Wanfang, and Weipu databases were searched from inception to June 15, 2021. The keywords were (Verburgh et al., 2014) exercise (physical activity, exercise, fitness, and sport) and (2) fNIRS (functional near-infrared spectroscopy). These terms were consistently applied across each database, serving as the main topic and free-text words in the title.

### 2.2 Eligibility criteria

Studies were considered eligible if they fulfilled the following criteria: (1) the subjects were healthy; (2) the articles were published in the English language or Chinese language in peer-reviewed journals; (3) exercise-related intervention studies utilizing large muscle groups of the whole body; and (4) at least one cerebral cortical blood flow change was assessed using fNIRS.

Our review focused on the effect of exercise interventions on common healthy participants. The exclusion criteria were as follows: unclear exercise protocols, exercise protocols not designed to improve brain or cognitive health (e.g., exercise test to exhaustion), and studies involving combined interventions (e.g., nutrition and cognition). To ensure generalizability, research utilizing clinical samples (e.g., overweight/obese) and those examining special groups (athletes or people with long-term exercise habits) were excluded.

### 2.3 Data extraction

Duplicated studies screened from the database search and reference lists were initially excluded. Next, the titles and abstracts were individually evaluated by two authors (J.M.H. and T.X.) to further exclude articles based on the eligibility criteria. Afterward, the two authors independently evaluated the articles. Disagreements were resolved by discussion and consensus among the three authors (Q.Q.S., J.M.H., and T.X.).

The two authors independently extracted the following data from eligible studies: (1) basic information, including the year of publication, participant characteristics, and study design; (2) study design, including study group or condition design, fNIRS state (resting-state or task-design), physiological outcome index, and behavioral outcome index; (3) fNIRS device parameters, including types of fNIRS devices, fNIRS sampling frequency, number of light emitting diodes, laser diodes, channels, fNIRS instrument location and area of interest, and position/arrangement and placement of the light source and detector; (4) the exercise intervention design, covering exercise type, exercise intervention period, frequency of exercise, exercise intensity, and single intervention duration; and (5) the primary endpoints of the studies.

### 2.4 Risk of bias assessment

**Figure 1:**
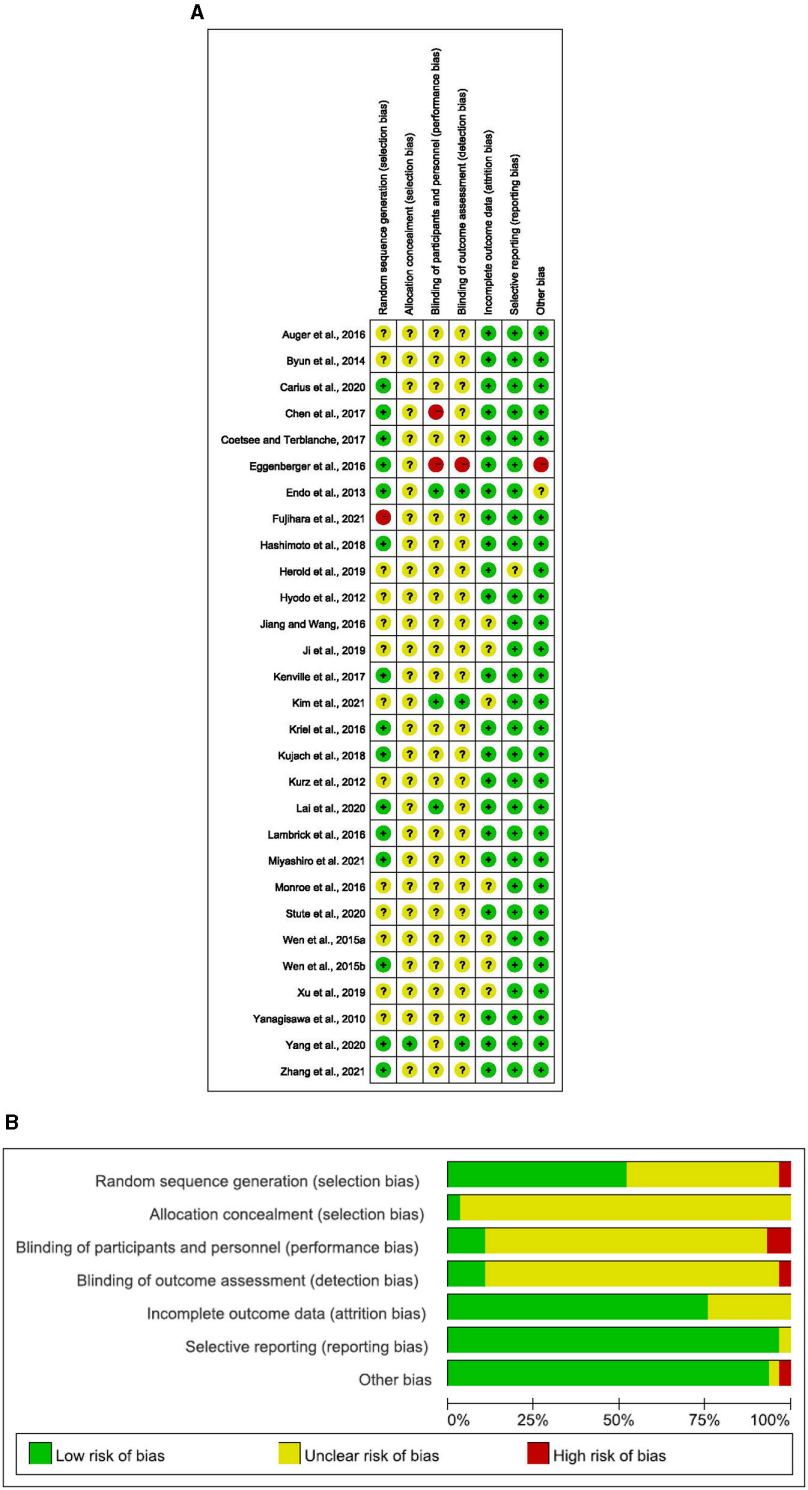
**(A)** Risk of bias ratings. **(B)** Risk of bias graph: percentage of trials with low, unclear, or high risk of bias ratings for each domain (Yanagisawa, 2010; Hyodo, 2012; Kurz et al., 2012; Endo et al., 2013; Byun et al., 2014; Wen et al., 2015a,b; Auger et al., 2016; Eggenberger et al., 2016; Jiang and Wang, 2016; Kriel et al., 2016; Lambrick et al., 2016; Monroe et al., 2016; Chen et al., 2017; Coetsee and Terblanche, 2017; Kenville et al., 2017; Hashimoto et al., 2018; Kujach, 2018; Herold et al., 2019; Ji et al., 2019; Xu et al., 2019; Carius et al., 2020; Lai et al., 2020; Stute et al., 2020; Yang et al., 2020; Kim et al., 2021; Miyashiro et al., 2021; Zhang et al., 2021).

The risk of bias in selected studies was independently assessed by two authors (J.M.H. and T.X.) using the Cochrane Collaboration Risk-of-Bias tool (Higgins et al., 2011, 2019). Disagreements were resolved by discussion with another author (Q.Q.S.) to achieve consensus (see Table 1 and Figure 1).

**Table 1:** Quality of included studies.

**References**	**Random sequence generation (selection bias)**	**Allocation concealment (selection bias)**	**Blinding of participants and personnel (performance bias)**	**Blinding of outcome assessment (detection bias)**	**Incomplete outcome data (attrition bias)**	**Selective reporting (reporting bias)**	**Other bias**
Auger et al. (2016)	U	U	U	U	L	L	L
Byun et al. (2014)	U	U	U	U	L	L	L
Carius et al. (2020)	L	U	U	U	L	L	L
Chen et al. (2017)	L	U	H	U	L	L	L
Coetsee and Terblanche (2017)	L	U	U	U	L	L	L
Endo et al. (2013)	L	U	L	L	L	L	U
Fujihara et al. (2021)	H	U	U	U	L	L	L
Hashimoto et al. (2018)	L	U	U	U	L	L	L
Eggenberger et al. (2016)	L	U	H	H	L	L	H
Herold et al. (2019)	U	U	U	U	L	U	L
Hyodo (2012)	U	U	U	U	L	L	L
Jiang and Wang (2016)	U	U	U	U	U	L	L
Ji et al. (2019)	U	U	U	U	U	L	L
Kenville et al. (2017)	L	U	U	U	L	L	L
Kim et al. (2021)	U	U	L	L	U	L	L
Kriel et al. (2016)	L	U	U	U	L	L	L
Kujach (2018)	L	U	U	U	L	L	L
Kurz et al. (2012)	U	U	U	U	L	L	L
Lai et al. (2020)	L	U	L	U	L	L	L
Lambrick et al. (2016)	L	U	U	U	L	L	L
Miyashiro et al. (2021)	L	U	U	U	L	L	L
Monroe et al. (2016)	U	U	U	U	U	L	L
Stute et al. (2020)	U	U	U	U	L	L	L
Wen et al. (2015a)	U	U	U	U	U	L	L
Wen et al. (2015b)	L	U	U	U	U	L	L
Xu et al. (2019)	U	U	U	U	U	L	L
Yanagisawa (2010)	U	U	U	U	L	L	L
Yang et al. (2020)	L	L	U	L	L	L	L
Zhang et al. (2021)	L	U	U	U	L	L	L

L, Low risk; H, High risk; U, Unclear risk.

## 3 Results

### 3.1 Study selection and characteristics

**Figure 2:**
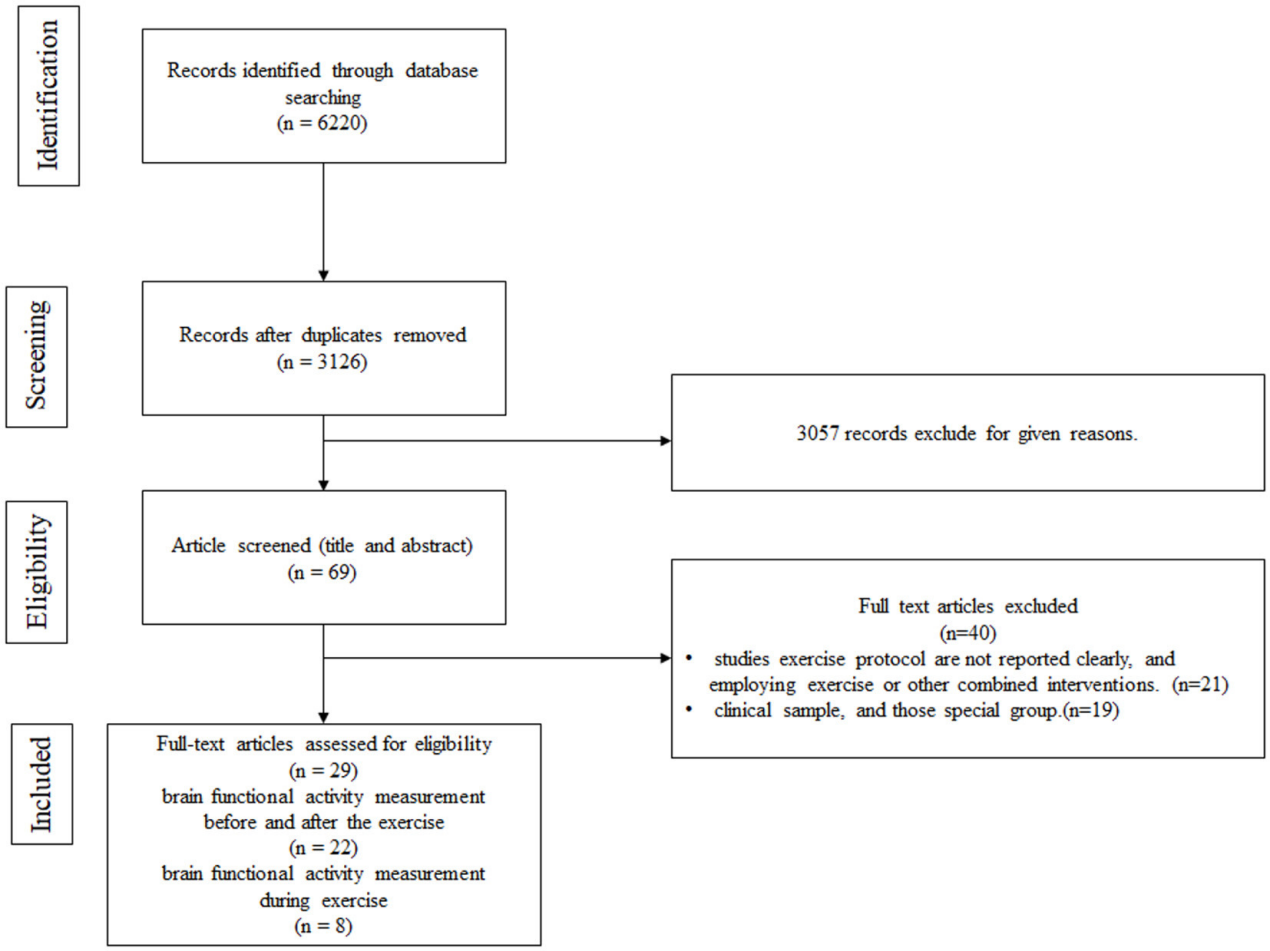
Flow chart.

The search process is detailed in a flow chart illustrated in Figure 2. The search strategy yielded 6,220 studies from the pre-defined databases. After excluding duplicates and reviewing the full text, 69 studies met the criteria based on the consensus reached by the reviewers. From these, 22 eligible articles were included in the first category (cerebral hemodynamics were measured before and after exercise) and 8 in the second category (cerebral hemodynamics were measured during exercise). Among them, one study was simultaneously in both categories.

Overall, 29 studies were included in the systematic review. Regarding the study region, 18 studies were conducted in Asia (Yanagisawa, 2010; Hyodo, 2012; Endo et al., 2013; Byun et al., 2014; Wen et al., 2015a,b; Jiang and Wang, 2016; Chen et al., 2017; Coetsee and Terblanche, 2017; Hashimoto et al., 2018; Kujach, 2018; Ji et al., 2019; Xu et al., 2019; Lai et al., 2020; Yang et al., 2020; Kim et al., 2021; Miyashiro et al., 2021; Zhang et al., 2021), 5 in Europe (Eggenberger et al., 2016; Kenville et al., 2017; Herold et al., 2019; Carius et al., 2020; Stute et al., 2020), 2 in Oceania (Kriel et al., 2016; Lambrick et al., 2016), 3 in North America (Kurz et al., 2012; Auger et al., 2016; Monroe et al., 2016), and 1 in Africa (Coetsee and Terblanche, 2017). A total of 664 participants were examined, with sample sizes ranging from 10 to 67. The age of patients across studies spanned from 72.3 months to 75 years.

### 3.2 Quality of included studies

The details on the quality of the included studies in bias risk assessment are summarized in the supporting material. Of note, 28 studies did not provide details on selective reporting, 27 studies reported no other biases, 23 studies reported complete outcome data, 15 studies reported random sequence generation, 1 study reported allocation concealment, 1 study reported blinding of participants and personnel, and 1 study reported blinding of outcome assessment.

### 3.3 Study design

Twenty-two studies measured cerebral hemodynamics before and after exercise interventions, eight studies (including only adults) documented cerebral hemodynamics during the exercise intervention, and one study recorded cerebral hemodynamics before, during, and after the exercise intervention.

#### 3.3.1 Study design encompassing measurements before and after exercise intervention

In this category (see Table 2), 22 studies provided information on cerebral hemodynamics and activity before and after exercise in the exercise group compared to levels measured before exercise in this group (14 studies) (Yanagisawa, 2010; Hyodo, 2012; Endo et al., 2013; Kujach, 2018; Miyashiro et al., 2021; Byun et al., 2014; Wen et al., 2015a,b; Jiang and Wang, 2016; Lambrick et al., 2016; Hashimoto et al., 2018; Ji et al., 2019; Xu et al., 2019; Kim et al., 2021) or cerebral hemodynamics levels measured before and after exercise in another group (8 studies) (Eggenberger et al., 2016; Chen et al., 2017; Coetsee and Terblanche, 2017; Lai et al., 2020; Stute et al., 2020; Yang et al., 2020; Fujihara et al., 2021; Zhang et al., 2021).

**Table 2:** Study design of measurement before and after exercise intervention.

**References**	**Age**	**Group or condition comparison**	**fNIRS state and task**	**Task design**	**Physiological outcome index**	**Behavioral outcome index**
Yanagisawa (2010)	21.5 ± 4.8	Ex condition; con condition	T ask state: Stroop task	Event-related	NA	NA
Hyodo (2012)	69.3 ± 3.5	Ex condition; con condition	Task state: Stroop task	Event-related	HR	RPE
Endo et al. (2013)	23 ± 1	20% EX_max_ condition; 40% EX_max_ condition; 60% EX_max_ condition; con condition	Resting-state: Sit for five min Task state: Stroop task, exercise task	NR	MAP; HR	RPE
Kujach (2018)	21	HIE condition; con condition	Task state: Stroop task	Event-related	HR	RPE; TDMS
Miyashiro et al. (2021)	20–24	Meditation condition; ex condition; con condition	Task state: N-back task	NR	NA	NA
Byun et al. (2014)	20.6 ± 1	Ex condition; con condition	Task state: Stroop task	Event-related	NA	RPE; TDMS
Wen et al. (2015a)	23.6 ± 1.2	Ex condition; con condition;	Task state: Flanker task	Block	HR	RPE
Wen et al. (2015b)	58.7 ± 7.2	Ex condition; con condition;	Task state: Flanker task	Block	HR	RPE
Eggenberger et al. (2016)	74.9 ± 6.9	Video game dance group; balance and stretching group	Task state: Walking task	Block	NA	TMT-A; TMT-B; Stroop task; Executive Control task; MoCA; SPPB; FES-I; GDS
Jiang and Wang (2016)	20.6	Ex condition;	Task state: Flanker task	Block	NA	NA
Lambrick et al. (2016)	8.8 ± 0.8	CONT condition; INT condition	Task state: Stroop task	NR	HR; VO_2_; V_E_; RER; energy expenditure	Eston-Parfitt Scale
Chen et al. (2017)	22.5 ± 2	BMB group; con group	Task state: Flanker task	Block	NA	POMS (short version)
Coetsee and Terblanche (2017)	62.7 ± 5.7	RT group; HIIT group; MCT group; con group	Task state: Stroop task	NR	Walking endurance	NA
Hashimoto et al. (2018)	24.3 ± 3.5	40% VO_2_ peak condition; 60% VO_2_ peak condition; con condition	Task state: Paced Auditory Serial Addition Test	Block	NA	NA
Ji et al. (2019)	65.6 ± 1.32	CE condition; PE condition; CE + PE condition; RC condition	Task state: Stroop task	Block	NA	NA
Xu et al. (2019)	20.7 ± 1.6	Exp condition; con condition	Task state: Table-setting task	Block	NA	NA
Lai et al. (2020)	Boys: 72.3 ± 2.74 months Girls: 73.2 ± 1.30 months	Tennis group; con group	Task state: N-back task	Block	NA	Physical fitness
Stute et al. (2020)	68.26 ± 3.31 69.7 ± 4.23	Exp group; con group	Task state: N-back task	Block	HR	RPE
Yang et al. (2020)	TCC Group: 66.31 ± 4.25 con Group: 65.92 ± 3.48	TCC group; con group	Task state: Flanker task	Block	NA	NA
Fujihara et al. (2021)	Young adults: 22.68 ± 1.38 Old adults: 68.72 ± 5.26	Young adult group; older adult group	Task state: Reverse Stroop task	NR	HRR	RPE
Kim et al. (2021)	22.5 ± 2.2	Moderate intensity condition; High-intensity condition	Task state: 2-back task	NR	NA	NA
Zhang et al. (2021)	Ex group: 22.10 ± 1.85 con group: 21.30 ± 2.54	Ex group; con group	Task state: Implicit cognitive reappraisal task	Block	NA	NA

HIE, high-intensity intermittent exercise; con, control; EX, exercise; HIIT, high-intensity interval training; RT, resistance training; MCT, moderate continuous training; EX_max_, maximum voluntary exercise; INT, intermittent bout of exercise; CE, cognitive exercise; PE, physical exercise; CE + PE, cognitive + physical exercise; RC, reading control; exp, experimental; CONT, continuous bout of exercise; NR, not reported; NA, not applicable.

Only one study measured hemodynamic changes and activity in the resting state. In this particular study, baseline brain activity was assessed in the seated position for 5 min (Endo et al., 2013).

All studies evaluated cortical hemodynamic activation using different task designs: 14 studies assessed inhibitory control (Flanker or Stroop task) (Yanagisawa, 2010; Hyodo, 2012; Endo et al., 2013; Byun et al., 2014; Wen et al., 2015a,b; Jiang and Wang, 2016; Lambrick et al., 2016; Chen et al., 2017; Coetsee and Terblanche, 2017; Kujach, 2018; Ji et al., 2019; Yang et al., 2020; Fujihara et al., 2021), 3 studies examined working memory (N-back task) (Lai et al., 2020; Stute et al., 2020; Kim et al., 2021), 1 study investigated attention (paced auditory serial addition test) (Hashimoto et al., 2018), 1 study assessed cognitive reappraisal (implicit cognitive reappraisal task) (Zhang et al., 2021), 1 study investigated the mirror neuron system (table-setting task) (Xu et al., 2019), one study applied a concentration task (2-back task) (Miyashiro et al., 2021), and one study assessed an exercise task (walking) (Eggenberger et al., 2016). Interestingly, the majority of designs were block designs used in 12 studies (Wen et al., 2015a,b; Eggenberger et al., 2016; Jiang and Wang, 2016; Chen et al., 2017; Hashimoto et al., 2018; Ji et al., 2019; Xu et al., 2019; Lai et al., 2020; Yang et al., 2020; Zhang et al., 2021), whereas an event-related design was applied in four studies (Yanagisawa, 2010; Hyodo, 2012; Byun et al., 2014; Kujach, 2018). The remaining studies did not report the study design.

Nine studies carried out physiological measurements after exercise, among which seven studies measured heart rate (HR) (Hyodo, 2012; Endo et al., 2013; Wen et al., 2015a,b; Lambrick et al., 2016; Kujach, 2018; Stute et al., 2020). Other physiological indicators, namely, heart rate reverse (HRR) (Fujihara et al., 2021), mean arterial blood pressure (MAP) (Endo et al., 2013), walking endurance (Coetsee and Terblanche, 2017), oxygen intake (VO_2_), minute ventilation (V_E_), respiratory exchange ratio (RER), and energy expenditure, were exclusively analyzed in one particular study (Lambrick et al., 2016).

Twelve studies investigated other behavioral indexes without examining cortical hemodynamic activation, among which eight studies measured Rating of Perceived Exertion (RPE) (Hyodo, 2012; Endo et al., 2013; Byun et al., 2014; Wen et al., 2015a,b; Kujach, 2018; Stute et al., 2020; Fujihara et al., 2021), and two studies measured Two-Dimensional Mood Scale (TDMS) (Byun et al., 2014; Kujach, 2018). Other behavioral indices, namely the Profile of Mood States (POMS) (short version) (Chen et al., 2017), physical fitness (Lai et al., 2020), Eston-Parfitt Scale (Lambrick et al., 2016), Trail Making Test Part A (TMT-A), Trail Making Test Part B (TMT-B), Stroop Word-Color Interference task, Executive Control task, Montreal Cognitive Assessment (MoCA), Short Physical Performance Battery (SPPB), Falls Efficacy Scale International (FES-I), and geriatric depression scale (GDS), were solely analyzed in one study (Eggenberger et al., 2016).

#### 3.3.2 Study design involving measurements during exercise interventions

In this category (see Table 3), eight studies presented data on cerebral hemodynamic activation during the exercise intervention. Of note, all studies used exercise tasks to investigate the task design. Most studies either used block designs or did not specify the design, whilst few studies provided a detailed description of the design of exercise tasks. As anticipated, studies adopting a block design employed relatively short durations for each block, similar to the cognitive task, ranging from 20 to 40 seconds.

**Table 3:** Study design of measurement during exercise interventions.

**References**	**Age**	**Group or condition**	**fNIRS state and task**	**Task design**	**Physiological outcome index**	**Behavioral outcome index**
Kurz et al. (2012)	23.7 ± 1.4	Walking forward condition; walking backward condition	Task state: walking	Block	NA	Stride time interval
Endo et al. (2013)	23 ± 1	20% EX_max_ condition; 40% EX_max_ condition; 60% EX_max_ condition; Control condition	Task state: cycling	NR	MAP; HR	RPE
Auger et al. (2016)	23.1 ± 2.4	40% POP condition; 80% POP condition; Rest condition;	Task state: cycling	Block	PPO	NA
Kriel et al. (2016)	23 ± 3	HIITPASS condition; HIITACT condition; REC condition	Task state: cycling	NR	VO_2_; HR; HHb; power output	NA
Monroe et al. (2016)	21.3 ± 2.4	SIC condition CRC condition	Task state: cycling	NR	HR; oxygen uptake; peak power	POMS-B; RPE
Kenville et al. (2017)	25.7 ± 2.2	BS with 0% 1 RM (L0%) condition; BS with 20% 1 RM (L20%) condition; BS with 40% 1 RM (L40%) condition; BL condition	Task state: barbell squat	Block	NA	NA
Herold et al. (2019)	25.00 ± 3.00	Overground condition; treadmill walking condition	Task state: walking	Block	HR; LF/HF ratio	Walking speed
Carius et al. (2020)	24.61 ± 0.47	DH_slow_ condition; NDH_slow_ condition; AH_slow_ condition; DH_fast_ condition; NDH_fast_ condition; AH_fast_ condition	Task state: basketball dribbling	Block	HR	VAS

DH_slow_, dominant right hand at slow walking pace; NDH_slow_, non-dominant left hand at slow walking pace; AH_slow_, alternating hands at slow walking pace; DH_fast_, dominant right hand at fast walking pace; NDH_fast_, non-dominant left hand fast walking pace; AH_fast_, alternating hands at fast walking pace; SIC, sprint interval cycling; CRC, constant resistance cycling; BS, barbell squat; HIITPASS, HIIT with passive recovery; HIITACT, HIIT with active recovery; REC, recovery; BL, baseline values; NR, not reported; NA, not applicable.

These exercise tasks, such as walking (Kurz et al., 2012; Herold et al., 2019), cycling (Endo et al., 2013; Auger et al., 2016; Kriel et al., 2016; Monroe et al., 2016), basketball slalom dribbling (Carius et al., 2020), and barbell squats (Kenville et al., 2017) were easy to perform in laboratory settings.

Furthermore, six studies conducted physiological measurements, of which five studies measured HR (Endo et al., 2013; Kriel et al., 2016; Monroe et al., 2016; Herold et al., 2019; Carius et al., 2020). Some physiological indicators, namely MAP (Endo et al., 2013), VO_2_ (Kriel et al., 2016), peak power output (PPO) (Auger et al., 2016), power output (Kriel et al., 2016), peak power (Monroe et al., 2016), oxygen uptake (Monroe et al., 2016), and LF/HF ratio (Herold et al., 2019), were only analyzed in one specific study.

Five studies investigated the effects of exercise on behavioral indices that only appeared in one particular study, namely stride time interval (Kurz et al., 2012), PRE (Endo et al., 2013; Monroe et al., 2016), Profile of Mood States-Brie (POMS-B) (Monroe et al., 2016), walking speed (Herold et al., 2019), and visual analog scale (VAS) (Carius et al., 2020).

### 3.4 fNIRS devices

#### 3.4.1 Measurements before and after exercise interventions

Most included studies conducted fNIRS tests before and after a single, long-term exercise intervention using eleven different fNIRS devices. The device sampling frequency ranged from 1 to 50 Hz, with the majority of devices utilizing 16 emitting diodes and 16 laser diodes. The number of channels ranged from 2 to 48.

Four studies focused on multiple cortical areas (Hashimoto et al., 2018; Xu et al., 2019; Stute et al., 2020). Among them, one study focused on motor areas, such as the premotor cortex (PMC) (Xu et al., 2019; Stute et al., 2020), whilst two studies reported findings on the parietal cortex (Xu et al., 2019; Stute et al., 2020), such as the inferior parietal cortex (IPC) and superior parietal lobule (SPL). Besides, two studies reported data on the prefrontal cortex (Stute et al., 2020; Yang et al., 2020), one study investigated temporal areas (Hashimoto et al., 2018), and one study assessed the inferior frontal gyrus (IFG). Nineteen studies exclusively assessed activation of the PFC (Yanagisawa, 2010; Hyodo, 2012; Endo et al., 2013; Byun et al., 2014; Wen et al., 2015a,b; Eggenberger et al., 2016; Jiang and Wang, 2016; Lambrick et al., 2016; Chen et al., 2017; Coetsee and Terblanche, 2017; Kujach, 2018; Ji et al., 2019; Lai et al., 2020; Yang et al., 2020; Fujihara et al., 2021; Kim et al., 2021; Miyashiro et al., 2021; Zhang et al., 2021). Details are listed in Table 4.

**Table 4:** The fNIRS devices used in the study design for measurement before and after exercise intervention.

**References**	**Instrument type**	**Sampling frequency**	**Diode**	**Laser**	**Channel**
Yanagisawa (2010)	ETG-7000	100 ms	16	16	48
Hyodo (2012)	ETG-7000	10 Hz	16	16	48
Endo et al. (2013)	NIRO 200	1 Hz	NR	NR	NR
Kujach (2018)	ETG-7000	10 Hz	8	8	48
Miyashiro et al. (2021)	ETG-4000	10 Hz	8	7	22
Byun et al. (2014)	ETG-7000	10 Hz	16	16	48
Wen et al. (2015a)	ETG-4000	10 Hz	16	16	44
Wen et al. (2015b)	ETG-4000	10 Hz	16	16	44
Eggenberger et al. (2016)	Oxiplex TS tissue spectrometer	1 Hz	8	2	NR
Jiang and Wang (2016)	NR	NR	NR	NR	NR
Lambrick et al. (2016)	PortaLite	2 Hz	3	3	NR
Chen et al. (2017)	ETG-4000	NR	6	10	44
Coetsee and Terblanche (2017)	NIRO 200NX	5 Hz	2	2	2
Hashimoto et al. (2018)	ETG-4000	100 ms	8	8	24
Ji et al. (2019)	NIRScout	3.91 Hz	8	8	20
Xu et al. (2019)	NIRSport	7.81 Hz	8	8	14
Lai et al. (2020)	PortaLite	50 Hz	3	1	3
Stute et al. (2020)	NIRSport	3.47 Hz	16	16	38
Yang et al. (2020)	ETG-4000	NR	16	14	44
Fujihara et al. (2021)	OEG-16	NR	6	6	16
Kim et al. (2021)	NIRSIT	8.138 Hz	24	32	48
Zhang et al. (2021)	OMM3000/8	33 Hz	9	9	27

#### 3.4.2 Measurements during exercise interventions

fNIRS was conducted during acute exercise interventions (see Table 5) using four distinct fNIRS devices were used. The device sampling frequency ranged from 1 to 1,000 Hz, and 8 emitting diodes and 8 laser diodes were employed in the majority of the studies. The number of channels ranged from 4 to 24.

**Table 5:** The fNIRS devices used in the study design for measurement during exercise intervention.

**References**	**Instrument type**	**Sampling frequency**	**Diode**	**Laser**	**Channel**
Kurz et al. (2012)	ETG-4000	10 Hz	8	8	24
Endo et al. (2013)	NIRO 200	1 Hz	NR	NR	NR
Auger et al. (2016)	Custom built	NR	4	4	NR
Kriel et al. (2016)	PortaLite	10 Hz	NR	NR	NR
Monroe et al. (2016)	OxyMon MKIII	1,000 Hz	2	2	4
Kenville et al. (2017)	NIRSport	7.81 Hz	8	8	NR
Herold et al. (2019)	NIRSport	7.81 Hz	8	8	14
Carius et al. (2020)	NIRSport	7.81 Hz	8	7	22

Four studies focused on multiple cortical areas (Kurz et al., 2012; Kenville et al., 2017; Herold et al., 2019; Carius et al., 2020). All studies focused on motor areas, such as the PMC, primary motor cortex (M1), supplementary motor area (SMA), and precentral gyrus (PCG) (Kurz et al., 2012; Kenville et al., 2017; Herold et al., 2019; Carius et al., 2020). Among them, three studies reported findings on the parietal cortex (Kurz et al., 2012; Kenville et al., 2017; Carius et al., 2020), such as the IPC and SPL, one study reported data on the PFC (Herold et al., 2019), one study assessed brain areas related to auditory, frontal and visual functions (Kurz et al., 2012; Kenville et al., 2017), including the primary somatosensory cortex (SSC) and, postcentral gyrus (POCG). Lastly, four studies reported data on PFC activation (Endo et al., 2013; Auger et al., 2016; Kriel et al., 2016; Monroe et al., 2016).

### 3.5 Exercise intervention

All exercise interventions were categorized into three types according to their frequency and duration, regardless of study design. In other words, they were measured before and after long-term exercise interventions (*n* = 5), measured before and after one-time exercise interventions (*n* = 17), and measured during one-time exercise interventions (*n* = 8). Among them, merely one study presented data before, during, and after acute exercise interventions. Major confounding factors adjusted for across these studies included exercise type, duration, intensity, frequency, and duration of activity.

#### 3.5.1 Design of measurements before and after exercise interventions

Five studies investigated hemodynamic changes before and after long-term exercise interventions. Since before-after tests were used, the influence of exercise on fNIRS imaging results was not considered. A broad range of exercise interventions was implemented in these studies, including walking (Coetsee and Terblanche, 2017), Tai Chi Chuan (TCC) (Yang et al., 2020), Baduanjin mind-body (BMB) (Chen et al., 2017), tennis (Lai et al., 2020) or interactive cognitive-motor video game dancing (DANCE), and balance and stretching training (BALANCE) (Eggenberger et al., 2016). The exercise intervention period lasted 8 weeks in most studies (Eggenberger et al., 2016; Chen et al., 2017; Lai et al., 2020; Yang et al., 2020), with only one study extending to 16 weeks (Coetsee and Terblanche, 2017). The frequency of exercise ranged from 2 to 5 times a week. Exercise intensity was classified into three categories: low, moderate, and high. Most studies employed moderate exercise intensity, except for one study that did not report data on intensity (Chen et al., 2017) and one that used moderate-vigorous (Coetsee and Terblanche, 2017) intensity. The duration of a single intervention ranged from 30 min to 90 min. Details are listed in Table 6.

**Table 6:** Exercise protocol used in the study design for measurement before and after long-term exercise intervention.

**References**	**Duration (weeks)**	**Sessions/week**	**Session length (min)**	**Exercise type**	**Exercise intensity**
Eggenberger et al. (2016)	8 weeks	3	30 min	DANCE or BALANCE	Moderate-vigorous
Chen et al. (2017)	8 weeks	5	90 min	BMB	NR
Coetsee and Terblanche (2017)	16 weeks	3	30 min	Walking	Moderate-vigorous
Lai et al. (2020)	8 weeks	2	60 min	Tennis	Moderate
Yang et al. (2020)	8 weeks	3	45 min	TCC	Moderate

NR, not reported.

Seventeen studies measured cerebral blood flow before and after acute exercise interventions (see Table 7). Exercise types involved cycling and running in the majority of studies, with the exception of one study that incorporated push-ups (Miyashiro et al., 2021). The duration of a single intervention varied from 10 min to 30 min, with seven studies employing a 10-min duration (Yanagisawa, 2010; Hyodo, 2012; Byun et al., 2014; Wen et al., 2015a,b; Kujach, 2018; Kim et al., 2021), five studies opting for 15 min (Endo et al., 2013; Hashimoto et al., 2018; Ji et al., 2019; Stute et al., 2020; Fujihara et al., 2021), two studies using a 20-min duration (Jiang and Wang, 2016; Miyashiro et al., 2021), one study implementing a duration of 25 min (Xu et al., 2019), and two studies extending to 30 min (Lambrick et al., 2016; Zhang et al., 2021). Lastly, exercise intensity was mostly moderate.

**Table 7:** Exercise protocol used in the study design for measurement before and after acute exercise intervention.

**References**	**Duration (weeks)**	**Sessions/week**	**Session length (min)**	**Exercise type**	**Exercise intensity**
Yanagisawa (2010)	NA	NA	10 min	Cycling	Moderate: 50% of VO_2_ peak
Hyodo (2012)	NA	NA	10 min	Cycling	Moderate: 50% of VO_2_ peak
Endo et al. (2013)	NA	NA	15 min	Cycling	20, 40, and 60% of EX_max_
Kujach (2018)	NA	NA	10 min	Cycling	60% of MAP
Miyashiro et al. (2021)	NA	NA	20 min	Push-ups	Not reported
Byun et al. (2014)	NA	NA	10 min	Cycling	Light: 30% of VO_2_ peak
Wen et al. (2015a)	NA	NA	10 min	Cycling	Moderate: 66% of HR_max_
Wen et al. (2015b)	NA	NA	10 min	Cycling	Moderate: 66% of HR_max_
Jiang and Wang (2016)	NA	NA	20 min	Cycling	Moderate intensity
Lambrick et al. (2016)	NA	NA	30 min	Running	Submaximal
Hashimoto et al. (2018)	NA	NA	15 min	Cycling	40% and 60% of the peak oxygen
Ji et al. (2019)	NA	NA	15 min	Cycling	65% of heart rate
Xu et al. (2019)	NA	NA	25 min	Cycling	Moderate intensity (65% of VO_2_ peak)
Stute et al. (2020)	NA	NA	15 min	Cycling	Moderate: 50% of VO_2_ peak
Fujihara et al. (2021)	NA	NA	15 min	Running	Moderate: HRR of 50%
Kim et al. (2021)	NA	NA	10 min	Running	Moderate-vigorous: 65% of VO_2_ peak-vigorous 80% of VO_2max_
Zhang et al. (2021)	NA	NA	30 min	Cycling	Moderate: 60%−69% of HR_max_

VO_2_ peak, peak oxygen intake; EXmax, maximum voluntary exercise; HRmax, maximal heart rate; VO_2_max, maximal oxygen intake; NA, not applicable.

#### 3.5.2 Design of measurements during acute exercise intervention

In the one-time exercise interventions, eight studies measured fNIRS during the exercise intervention (see Table 8). These studies mainly selected exercise interventions involving minimal head movement, such as cycling (Endo et al., 2013; Auger et al., 2016; Kriel et al., 2016; Monroe et al., 2016), basketball slalom dribbling (Carius et al., 2020), barbell squats (Kenville et al., 2017), and walking (Kurz et al., 2012; Herold et al., 2019). Moreover, most studies implemented cycling and walking interventions, while four studies used cycling (Endo et al., 2013; Auger et al., 2016; Kriel et al., 2016; Monroe et al., 2016), and two studies used walking (Kurz et al., 2012; Herold et al., 2019). The duration of the intervention ranged from 10 min to 25 min. While moderate intensity was used in most of the eight studies, some studies did not report exercise intensity and instead reported data on the exercise load.

**Table 8:** Exercise protocol used in the study design measurement during acute exercise intervention.

**References**	**Duration (weeks)**	**Sessions/week**	**Session length (min)**	**Exercise type**	**Exercise intensity**
Kurz et al. (2012)	NA	NA	20 min	Walking	Walking at 0.45 m/s
Endo et al. (2013)	NA	NA	15 min	Cycling	20%, 40%, and 60% of EX_max_
Auger et al. (2016)	NA	NA	10 min	Cycling	A constant power representing 40% and 80% of their individual PPO
Kriel et al. (2016)	NA	NA	18 min	Cycling	Vigorous
Monroe et al. (2016)	NA	NA	18 min	Cycling	The SIC of each 30-s sprint was summed across the four sprints to calculate the total work performed (Work SIC) to match the CRC at 70 rpm
Kenville et al. (2017)	NA	NA	25 min	Barbell load	0% 1 RM, 20% 1 RM, and 40% 1 RM for a BS
Herold et al. (2019)	NA	NA	~14.7 min	Walking	NR
Carius et al. (2020)	NA	NA	12.6 min	Basketball slalom dribbling	Slow walking pace (0.87 ms^−1^) and fast walking pace (1.75 ms^−1^)

EX_max_, maximum voluntary exercise; PPO, peak power output; 1RM, 1 repetition maximum; SIC, sprint interval cycling; CRC, constant resistance cycling; NA, not applicable; NR, not reported.

### 3.6 Main results

A total of 29 studies investigated oxyhemoglobin (oxy-Hb), deoxyhemoglobin (deoxy-Hb), and total hemoglobin (total Hb) levels following exercise interventions. Specifically, three studies measured oxy-Hb, deoxy-Hb, and total Hb levels (Auger et al., 2016; Lambrick et al., 2016; Coetsee and Terblanche, 2017), eight studies measured oxy-Hb and deoxy-Hb levels (Hyodo, 2012; Kurz et al., 2012; Endo et al., 2013; Byun et al., 2014; Monroe et al., 2016; Kenville et al., 2017; Herold et al., 2019; Carius et al., 2020), 16 studies measured oxy-Hb levels (Yanagisawa, 2010; Wen et al., 2015a,b; Eggenberger et al., 2016; Jiang and Wang, 2016; Chen et al., 2017; Hashimoto et al., 2018; Kujach, 2018; Ji et al., 2019; Xu et al., 2019; Lai et al., 2020; Yang et al., 2020; Fujihara et al., 2021; Kim et al., 2021; Miyashiro et al., 2021; Zhang et al., 2021), one study measured deoxy-Hb levels (Kriel et al., 2016), and one study computed the HBdiff (oxy-Hb minus deoxy-Hb) (Stute et al., 2020).

#### 3.6.1 Changes in brain functional activity before and after exercise interventions

Five studies investigated cerebral blood flow after long-term exercise interventions (see Table 9). One study measured oxy-Hb, deoxy-Hb, and total Hb levels (Coetsee and Terblanche, 2017), whilst the remaining four studies measured oxy-Hb levels (Eggenberger et al., 2016; Chen et al., 2017; Lai et al., 2020; Yang et al., 2020). After the long-term intervention, oxy-Hb levels were increased in the left PFC during the flanker and N-back tasks. Likewise, deoxy-Hb levels were increased in the left PFC during the Stroop task across almost all studies. One study used a walking task and described that oxy-Hb levels were higher in the left PFC and right PFC during walking.

**Table 9:** Changes of brain functional activity before and after long-term exercise interventions.

**References**	**Study design 1. Task 2. Oxygenation index**	**Exercise intensity**	**Region of interest (ROI)**	**Results**
Coetsee and Terblanche (2017)	1. Stroop task (naming condition) 2. oxy-Hb; deoxy-Hb; total Hb	Moderate-vigorous	Bilateral PFC	**Pre vs. Post** **Con group:**↑ left PFC (oxy-Hb) **MCT group:** ↑ left PFC (deoxy-Hb); ↓ left PFC (total Hb) **Con group vs. HIIT group, MCT group, and RT group**: **post:** ↓left PFC, significant difference (oxy-Hb)
	1. Stroop task (executive condition) 2. oxy-Hb; deoxy-Hb; total Hb		Bilateral PFC	**Pre vs. Post** **RT group:**↓ left PFC (oxy-Hb), left PFC (total Hb);↑ left PFC (deoxy-Hb) **MCT group:** ↑left PFC (deoxy-Hb); ↓left PFC (total Hb) **Con group vs. HIIT group and MCT group:** post: ↓left PFC, significant difference (oxy-Hb)
Chen et al. (2017)	1. Flanker task 2. oxy-Hb	NR	Bilateral PFC	**Pre vs. Post** **Baduanjin intervention group:**↑ left PFC
Yang et al. (2020)	1. Flanker task 2. oxy-Hb	Moderate	Frontal_Sup_L, Frontal_Inf_L, Frontal_Sup_R, rontal_Inf_R.	**Pre vs. Post** **TCC group:**↑ Frontal_Sup_L, Frontal_Inf_L
Lai et al. (2020)	1.1-back task 2. oxy-Hb	Moderate	Left PFC	**Pre vs. Post** **Tennis intervention group:** ↑ left PFC **Tennis intervention group (**Time segment 1, 2, 3): ↑ left PFC
Eggenberger et al. (2016)	1. Walking task 2. oxy-Hb	Moderate-vigorous	Bilateral PFC	**Pre vs. Post** **Preferred t1-7:** ↓ left PFC, right PFC **Fast walking:** ↓ left PFC **Preferred vs. Fast Walking Speeds t1-7** **Post:** ↓ right PFC **Preferred vs. Fast Walking Speeds t10-25** **Post** ↓ left PFC **Baseline vs. Experimental** **Preferred and Fast Walking Speeds t1-7, t10-25, t26-34**, ↓ left PFC, right PFC **Referred and Fast Walking Speeds t35-46:** ↑ left PFC, right PFC

pre, pretest; post, posttest; ex, exercise; con, control; HIIT, high-intensity interval training; MCT, moderate continuous training; RT, resistance training; t1–7, The first timeframe was set at the initiation and acceleration of walking from the first to the seventh s of walking (t1–7); t10–25, the second timeframe t10–25 represented “steady-state” walking; t26–34, the end of walking with deceleration; t35-46 was chosen to analyze the decrease in oxy-Hb levels below the baseline during the rest phases. ↑, compared with the former, the latter was higher than the former; ↓, compared with the former, the latter was lower than the latter. Nonsignificant changes in brain activity were not reported. Frontal_Sup_L: the frontal superior left area, Frontal_Inf_L: the frontal inferior left area, Frontal_Sup_R: the frontal superior right area, Frontal_Inf_R: the frontal inferior area right.

Seventeen studies investigated brain function before and after the acute exercise intervention (see Table 10). One study measured oxy-Hb, deoxy-Hb, and total Hb levels (Lambrick et al., 2016), three studies analyzed oxy-Hb and deoxy-Hb levels (Yanagisawa, 2010; Endo et al., 2013; Miyashiro et al., 2021), 12 studies measured oxy-Hb levels (Yanagisawa, 2010; Wen et al., 2015a,b; Jiang and Wang, 2016; Hashimoto et al., 2018; Kujach, 2018; Ji et al., 2019; Xu et al., 2019; Fujihara et al., 2021; Kim et al., 2021; Miyashiro et al., 2021; Zhang et al., 2021), and one study calculated the HBdiff (oxy-Hb minus deoxy-Hb) (Herold et al., 2019). After the acute intervention, eight articles explored changes in the Stroop task (Yanagisawa, 2010; Hyodo, 2012; Endo et al., 2013; Byun et al., 2014; Lambrick et al., 2016; Kujach, 2018; Ji et al., 2019; Fujihara et al., 2021) and observed an increase in oxy-Hb levels in the left dorsolateral prefrontal cortex (DLPFC) (Yanagisawa, 2010; Hyodo, 2012; Byun et al., 2014; Kujach, 2018; Ji et al., 2019), bilateral PFC (Endo et al., 2013), right frontopolar area (FPA) (Hyodo, 2012), left FPA (Byun et al., 2014), middle PFC (Fujihara et al., 2021), right ventrolateral prefrontal cortex (VLPFC) (Ji et al., 2019), and supraorbital ridge of the dominant side (Lambrick et al., 2016). Meanwhile, three articles explored changes in flanker task performance and observed that exercise resulted in an increase in oxy-Hb levels in the bilateral DLPFC (Wen et al., 2015b), right DLPFC, right FPA (Wen et al., 2015a), and left FPA (Wen et al., 2015b). Similarly, three articles explored fluctuations in performance on the n-back task and noted a rise in oxy-Hb levels in the bilateral orbitofrontal cortex (OFC) (Miyashiro et al., 2021) and left DLPFC (Kim et al., 2021) and a concomitant decrease in oxy-Hb levels in the right DLPFC during moderate-intensity exercise interventions (Kim et al., 2021), whilst HBdiff was decreased in both regions (frontal and parietal) and hemispheres (left and right) at almost all time points (Stute et al., 2020). One study applied the table-setting task (Xu et al., 2019) and found elevated oxy-Hb levels in the PMC, SPL, inferior frontal gyrus (IFG), and rostral inferior parietal lobule (IPL). Another study used the Paced Auditory Serial Addition Test (Hashimoto et al., 2018) and revealed that oxy-Hb levels in the left PFC increased with different exercise intensities. Finally, one study used the implicit cognitive reappraisal task but did not identify specific regions of interest (ROIs) with changes in activity after exercise and reported elevated oxy-Hb levels in channels 11, 16, 21, 23, and 27 (Zhang et al., 2021).

**Table 10:** Changes of brain functional activity before and after acute exercise interventions.

**References**	**Study design 1. Task 2. Oxygenation index**	**Exercise intensity**	**Region of interest (ROI)**	**Results**
Yanagisawa (2010)	1. Stroop task 2. oxy-Hb	Moderate: 50% of VO_2_ peak	Anterior VLPFC; left DLPFC; left FPA; right DLPFC	**Pre vs. Post** **ex group and con group:** ↑ left DLPFC
Hyodo (2012)	1. Stroop task 2. oxy-Hb; deoxy-Hb	Moderate: 50% of VO_2_ peak	Bilateral DLPFC, VLPFC, FPA	**Con group vs. ex group** **Pre:** ↑ bilateral DLPFC, VLPFC, FPA (oxy-Hb) ↑ DLPFC (deoxy-Hb) **Post:** ↑ right FPA (oxy-Hb)
Endo et al. (2013)	1. Stroop task 2. oxy-Hb; deoxy-Hb	20%, 40%, and 60% of EX_max_	Bilateral PFC	**Baseline vs. during Stroop 1 (before exercise)** **Without exercise:** ↑ bilateral PFC (oxy-Hb) **20% EX_max_,** **40% EX_max_,** **60% EX**_**max**_**:** ↑ bilateral PFC (oxy-Hb) **Baseline vs. during Stroop 2 (after exercise)** **40% EX**_**max**_**:** ↑ bilateral PFC (oxy-Hb) **Pre vs. Post** **40% EX**_**max**_**:** ↑ bilateral PFC (oxy-Hb) **60% EX**_**max**_**:** ↑ bilateral PFC (oxy-Hb) **Without exercise vs. 40% EX**_**max**_ **after exercise:** ↑ bilateral PFC (oxy-Hb) **Without exercise vs. 60% EX**_**max**_ **after exercise:** ↑ bilateral PFC (oxy-Hb)
Kujach (2018)	1. Stroop task 2. oxy-Hb	60% of MAP	Bilateral DLPFC, VLPFC, FPA	**Resting control vs. HIE** **Post:** ↑ left DLPFC **Pre vs. Post** **HIE:** ↑ left DLPFC **resting control:** ↑ right VLPFC
Byun et al. (2014)	1. Stroop task 2. oxy-Hb; deoxy-Hb	Light: 30% of VO_2_ peak	Bilateral DLPFC, VLPFC, FPA	**Con group vs. ex group** **Post:** ↑ left DLPFC (oxy-Hb) **Post:** ↑ left FPA (oxy-Hb)
Lambrick et al. (2016)	1. Stroop task 2. oxy-Hb; deoxy-Hb; total Hb	Submaximal	The supraorbital ridge of the participant's dominant side	**Pre vs. 1 min_post** **CONT and INT:** ↑ supraorbital ridge of the dominant side (oxy-Hb), supraorbital ridge of the dominant side (total Hb) **1 min_post vs. 15 min_post** **CONT and INT:** ↓ supraorbital ridge of the dominant side (oxy-Hb), supraorbital ridge of the dominant side (Total-Hb) **Pre vs. 15 min_post** **CONT and INT:** ↑ supraorbital ridge of the dominant side (oxy-Hb), supraorbital ridge of the dominant side (total Hb) **Pre vs. 30 min_post** **CONT and INT:** ↑ supraorbital ridge of the dominant side (oxy-Hb), supraorbital ridge of the dominant side (total Hb) **1 min_post vs. 30 min_post** **CONT) and INT:** ↓supraorbital ridge of the dominant side (deoxy-Hb)
Ji et al. (2019)	1. Stroop task (naming condition) 2. oxy-Hb	65% of heart rate	Bilateral VLPFC, and DLPFC	**RC condition vs. PE condition** ↑ right VLPFC
	1. Stroop task (executive conditions) 2. oxy-Hb		Bilateral VLPFC, and DLPFC	**RC condition vs. PE condition** ↑ left DLPFC, right DLPFC **CE condition vs. PE condition** ↑ right DLPFC
Fujihara et al. (2021)	1. Stroop task 2. oxy-Hb	Moderate: HRR of 50%	The left, middle, and right PFC	**Young adult group vs. older adult group** **Post:**↑ M-PFC
Wen et al. (2015a)	1. Flanker task 2. oxy-Hb	Moderate: 66% of HR_max_	Bilateral DLFPC, FPA, VLFPC	**Con group vs. ex group** **Post:**↑ left PA
Wen et al. (2015b)	1. fFanker task 2. oxy-Hb	Moderate: 66% of HR_max_	Bilateral DLFPC, FPA, VLFPC	**Con group vs. ex group** **Post:**↑ right DLPFC, right FPA
Jiang and Wang (2016)	1. Flanker task 2. oxy-Hb	Moderate intensity	Bilateral frontal area	**Pre vs. Post** **20 min of moderate-intensity aerobic ex group:** ↑ Bilateral frontal area
Miyashiro et al. (2021)	1.2-back task 2. oxy-Hb	Not reported	DLPFC and OFC, Frontopolar prefrontalCortex	**Meditation-control pair and the exercise-control pair vs. the meditation-exercise** **Post:**↑ right and left OFC
Stute et al. (2020)	1. N-back task 2. HBdiff	Moderate: 50% of VO_2_ peak	Bilateral DLPFC and VLPFC, IPL and SPL	**Con group vs. exp group** ↓ both regions (frontal and parietal) and hemispheres (left and right) at almost all time points
Kim et al. (2021)	1.2-back task 2. oxy-Hb	Moderate-vigorous: 65% of VO_2_ peak-vigorous 80% of VO_2max_	Bilateral DLPFC, OFC	**Pre vs. Post** **Moderate Intensity:** ↓ right DLPFC **High Intensity:** ↑ left DLPFC
Hashimoto et al. (2018)	1. Paced auditory serial addition test 2. oxy-Hb	40% and 60% of the peak oxygen	Left frontal and temporal areas	**40% VO**_**2**_ **peak and rest vs. 60% VO**_**2**_ **peak** **Post:** ↑ left PFC
Xu et al. (2019)	1. Table-setting task 2. oxy-Hb	Moderate intensity (65% of VO_2_ peak)	Left IFG, PMC, rostral IPL, and SPL	**Activation Pattern Assessed Using Channel-Based Group Analysis** **exec and obs components in the post-exp condition:** Activated in all channels (left IFG, PMC, SPL, rostral IPL) **Post-exp-obs condition:** Activated in channels 2, 6, 9, 11, and 13 (left IFG, PMC, and SPL) **Post-exp-obs components in the no-exercise conditions (post-ctrl, pre-exp, and pre-ctrl conditions):** Channels 3, 4, 5, 6, 7, 9, 11, 12, 13, and 14 (left IFG, PMC, rostral IPL, and SPL). According to the spatial map of the 23 subjects in the pre-exp condition and pre-ctrl condition, also found that channels 2, 3, 4, 5, 6, 7, 9, 10, and 13 (IFG, PMC, and rostral IPL), were activated during both action execution and observation. **Activation pattern assessed using ROI-based group analysis** **Action observation and execution in the post-exp condition:** Activated in left IFG, PMC, rostral IPL, and SPL, except SPL during action execution. **Action observation and execution in all the no-exercise conditions:** the IFG, PMC, rostral IPL, and SPL were significantly activated. Only the activation of the SPL during action execution in the post-ctrl condition and the rostral IPL during action execution in the post-ctrl condition were not significantly activated **ROI-based group analysis for the effect of moderate-intensity exercise** **In action observation, during the post-sessions (exp/ctrl)**, there were significant differences between the exp and ctrl conditions in all four ROIs. **In action execution, post-ctrl vs. post-exp:** ↑left IFG
Zhang et al. (2021)	1. Implicit cognitive reappraisal task 2. oxy-Hb	Moderate: 60%−69% of HR_max_	NR	**Con group vs. ex group** ↓ channels 1, 25 **Baseline vs. Implicit cognitive reappraisal task** ↓ channels 3 and 8 ↑ channels 11, 16, 21, 23, and 27 **Pre vs. Post** **ex group:** ↑ channels 7 and 13

NR, not reported; pre, pretest; post, posttest; ex, exercise; con, control; exp, experimental; EX_max_, maximum voluntary exercise; HIE, high-intensity intermittent exercise; CONT, an acute bout of continuous exercise; INT, an acute bout of intermittent exercise; PE, physical exercise; CE, cognitive exercise; exec, action execution; obsc, action observation.↑, compared with the former, the latter was higher than the former; ↓, compared with the former, the latter was lower than the former. Nonsignificant changes in brain activity were not reported.

#### 3.6.2 Changes in brain functional activity during exercise interventions

Eight studies investigated hemodynamic changes during exercise interventions (see Table 11). Studies using a one-time exercise intervention measured cerebral blood flow during exercise (Endo et al., 2013). Six studies analyzed both oxy-Hb and deoxy-Hb levels (Kurz et al., 2012; Endo et al., 2013; Monroe et al., 2016; Kenville et al., 2017; Herold et al., 2019; Carius et al., 2020), one study exclusively analyzed oxy-Hb, deoxy-Hb, and total Hb levels (Auger et al., 2016), and one study detected deoxy-Hb levels (Kriel et al., 2016).

**Table 11:** Changes of brain functional activity during acute exercise interventions.

**References**	**Study design 1. Task 2. Oxygenation index**	**Exercise intensity**	**Region of interest (ROI)**	**Results**
Kurz et al. (2012)	1. Walking task 2. oxy-Hb; deoxy-Hb	Walking at 0.45 m/s	SMA, SPL, precentral gyrus (PCG), postcentral gyrus (POCG)	**Forwards vs. backward walking** ↑SMA, PCG, SPL (oxy-Hb), ↓SMA (deoxy-Hb)
Endo et al. (2013)	1. Cycling taskv 2. oxy-Hb; deoxy-Hb	20%, 40%, and 60% of EX_max_	Bilateral PFC	**Baseline vs. during cycling task** **60% EX**_**max**_**:** ↑ bilateral PFC (oxy-Hb) **Without exercise vs. 60% EX**_**max:**_ ↑ bilateral PFC (oxy-Hb)
Auger et al. (2016)	1. Cycling task 2. oxy-Hb; deoxy-Hb; total-Hb	A constant power representing 40% and 80% of their individual PPO	PFC	**Rest vs. 80%PPO** ↑ PFC (deoxy-Hb)
Kriel et al. (2016)	1. Cycling task 2. Δdeoxy-Hb	Vigorous	PFC	**HIITPASS and HIITACT and REC** For the mean Δdeoxy-Hb for each bout, differences were found between conditions for Bout 2, Bout 3, and Bout 4; however, no significant differences in PFC were found between the two HIIT conditions. For the mean Δdeoxy-Hb within conditions, there were significant increases across bouts, with values increasing over time in the HIITPASS and HIITACT conditions.
Monroe et al. (2016)	1. Cycling task 2. Oxy-Hb; Deoxy-Hb	The SIC of each 30-s sprint was summed across the four sprints to calculate the total work performed (Work SIC) to match the CRC at 70 rpm	DLPFC	**Baseline vs. exercise** ↑DLPFC(Oxy-Hb) **CRC vs. SIC** ↑DLPFC(Oxy-Hb) **During SIC:** ↑DLPFC (Deoxy-Hb)
Kenville et al. (2017)	1. Barbell squat task 2. oxy-Hb; deoxy-Hb	0% 1RM, 20% 1RM, and 40% 1RM for a BS	M1, PMC, SMA, IPL, SPL, and brain areas that are related to auditory, frontal, and visual areas	**Alterations in the haemodynamic response within sensorimotor areas:** **L0% vs. L20%** ↑ bilateral M1, right PMC, SMA, left IPL, right SPL (oxy-Hb) **L0% vs. L40%** ↑ significant alterations in the hemodynamic response in all channels, except channels 12, 18, and 22 (oxy-Hb) ↑ left SSC and IPL (deoxy-Hb) **L20% vs. L40%** ↑ significant alterations in the hemodynamic response in all channels, except channels 4, 9, 12, and 18 (oxy-Hb) **BL vs. BS during L0%** ↑ bilateral M1, SSC, SMA, SPL, left IPL, and right PMC (oxy-Hb) **BL vs. BS during L20% and L40%** ↑ bilateral M1, SSC, SMA, SPL, left IPL, and right PMC (oxy-Hb)
				**Alterations in the haemodynamic response outside sensorimotor areas:** **L0% vs. L20%** ↑ bilateral M1, IPL, SMA, and PMC (oxy-Hb) **L0% vs. L40%** ↑ significant alterations in the hemodynamic response in all channels, except channels 1, 12, and 13 (oxy-Hb) **L20% vs. L40%** ↑ significant alterations in the hemodynamic response in all channels, except channels 1 and 13 (Oxy-Hb) **BL vs. BS during L0%, L20% and L40%** significant alterations in the hemodynamic response in terms of HbO_2_ levels in all fNIRS channels, except channels 1 and 13 (oxy-Hb)
				**Alterations in the haemodynamic response within sensorimotor areas after Short Distance Channel Regression:** **L0% vs. L40%** ↑ bilateral SPL (oxy-Hb) **L20% vs. L40%** ↑ bilateral SPL (oxy-Hb) ↑ left PMC (deoxy-Hb) **BL vs. L40%** ↑ left PMC (deoxy-Hb)
Herold et al. (2019)	1. Walking task 2. oxy-Hb; deoxy-Hb	NR	Bilateral PFC, PMC, SMA	**During walking** ↑left PFC, right PFC, left PMC (oxy-Hb) **Overground vs. treadmill walking** ↑ left PFC, right PFC, left PMC, right PMC, bilateral SMA (oxy-Hb)
Carius et al. (2020)	1. Basketball dribbling task 2. oxy-Hb; deoxy-Hb	Slow walking pace (0.87 ms^−1^) and fast walking pace (1.75 ms^−1^)	Bilateral PMC-SMA, SAC, M1, IPC	**AH**_**slow**_ **vs. DH**_**slow**_ ↓ IL-M1 (deoxy-Hb) **slow vs. fast** **dominant right hand (DH):** ↓ contralateral PMC-SMA (deoxy-Hb)

PMC-SMA, premotor and supplementary motor cortex; SAC, somatosensory association cortex; SSC, primary somatosensory cortex; IL-M1, ipsilateral M1; pre, pretest; post, posttest; Bout (every 30 seconds of high-intensity exercise is called a bout); L0%, 0% 1 RM (repetition maximum); EX_max_, maximum voluntary exercise; BS, barbell squat; BL, baseline values; HIITPASS, HIIT with passive recovery periods between four bouts; HIITACT, HIIT with active recovery periods between four bouts; REC, HIITACT with four HIIT bouts replaced with passive periods; FH, the prefrontal cerebral cortex; AH, alternating hands; DH, dominant right hand; AH_slow_, slow alternating hands; DH_slow_, slow dominant right hand; SIC, sprint interval cycling; CRC, constant resistance cycling. ↑, compared with the former, the latter was higher than the former; ↓, compared with the former, the latter was lower than the former. Nonsignificant changes in brain activity were not reported.

During the acute intervention, eight studies explored changes in various exercise conditions. Three studies explored changes in different cycling intensities and noted that oxy-Hb levels were increased in bilateral PFC during exercise at the intensity of 60% EX_max_ (Endo et al., 2013) and that under all three conditions of rest, 40%, and 80% intensity levels. The subjects at rest exhibited significantly lower extracerebral and cerebral deoxy-Hb levels compared to values measured at the 80% intensity level of exercise. Furthermore, another study detected significant alterations in the hemodynamic response in almost all channels, with increased oxy-Hb in the bilateral SPL and left PMC after short-distance channel regression (Kenville et al., 2017). At the same time, two studies explored the effects of different levels of exercise difficulties on cerebral hemodynamics. Four studies varied exercise intensity and found an increase in oxy-Hb levels in the bilateral PFC, bilateral M1, SSC, SMA, SPL, left IPL, and right PMC and a concurrent decrease in deoxy-Hb levels in the PFC. One study compared forward walking with backward walking and documented that oxy-Hb levels were higher in the SMA, PCG, and SPL, whereas deoxy-Hb levels were decreased in the SMA. Another study compared brain function in overground and treadmill conditions and observed an increase in oxy-Hb levels in the L-PFC, R-PFC, L-PMC, R-PMC, and B-SMA. One study explored the effect of active recovery on brain function and detected an increase in PFC activity. An earlier study employed BSDT to explore brain function under different basketball dribbling conditions and found that IL-M1 (deoxy-Hb levels) and contralateral PMC-SMA (deoxy-Hb levels) activities were decreased. One study investigated the effect of active recovery on brain function and evinced a significant interaction between condition and bout (every 30 seconds of high-intensity exercise is termed a bout) for mean changes in Δ[HHb] across bouts. Within conditions, significant increases in mean Δ[HHb] were observed across bouts, with values progressively increasing over time under HIITPASS and HIITACT conditions.

## 4 Discussion

### 4.1 Study design

Most task-related fNIRS studies aimed to detect activation before and after exercise. Notably, relatively few studies have been conducted on resting-state hemodynamics in this field; only one study examined changes in resting-state hemodynamics before and after exercise (Endo et al., 2013) and did not identify significant changes. Studies performing measurements before and after exercise interventions provide evidence of the effects of exercise on an individual's neural mechanisms.

A minority of the included studies explored cerebral blood flow during exercise interventions (Kurz et al., 2012; Endo et al., 2013; Auger et al., 2016; Kriel et al., 2016; Monroe et al., 2016; Kenville et al., 2017; Herold et al., 2019; Carius et al., 2020), all of which were task-designed and used exercise type as an exercise task. Nevertheless, it is worth mentioning that there are limited available exercise-type options, and they are relatively fixed. Most exercise tasks in those studies adopted the same design as cognitive tasks and controlled the duration of each trial to approximately 30 to 40 seconds (Kurz et al., 2012; Kenville et al., 2017; Carius et al., 2020). However, from a practical perspective, exercise tasks lasting <30 seconds are challenging to implement. Thus, fNIRS should be implemented to develop an exercise task that combines the characteristics of the movement with the feasibility of fNIRS, and suitable methods should be selected to analyze the fNIRS data. Notably, although fNIRS is not as sensitive to motion artifacts as functional MRI and EEG, the rapid motion of any vibrating fiber may lead to substantial changes in the hemoglobin signal. Therefore, when designing exercise tasks, frequent and intense head movements should be avoided.

In addition, numerous studies have explored the effects of exercise interventions on behavioral and physiological indicators before, during, and after exercise. Most of the behavioral indicators are related to cognition (Yanagisawa, 2010; Hyodo, 2012; Endo et al., 2013; Byun et al., 2014; Wen et al., 2015a,b; Jiang and Wang, 2016; Lambrick et al., 2016; Chen et al., 2017; Coetsee and Terblanche, 2017; Hashimoto et al., 2018; Kujach, 2018; Ji et al., 2019; Xu et al., 2019; Lai et al., 2020; Yang et al., 2020; Fujihara et al., 2021; Kim et al., 2021; Miyashiro et al., 2021) and emotion (Zhang et al., 2021), while the primary physiological indicator is heart rate, which is closely related to exercise. However, studies examining the relationship between functional brain activity and the three behavioral and physiological domains were scarce.

Future studies can combine real-time changes in cerebral blood flow before, during, and after exercise in a more in-depth and comprehensive manner to establish the relationship between exercise and brain plasticity. In addition, the benefits and mechanisms of exercise interventions on the health of individuals should be explored from multiple perspectives, combining behavioral, physiological, and cerebral assessments.

### 4.2 fNIRS equipment and parameter settings

The included studies measured cerebral hemodynamics before and after exercise employing a relatively large number of channels. In studies designed to measure cerebral hemodynamics during exercise, the number of channels was relatively low, with the maximum number of channels in the included studies being 24 (Kurz et al., 2012). Attributed to its task specificity, studies that measured cerebral hemodynamics during exercise intervention all used portable devices.

Besides, the selection of ROIs also varied with the study design. Studies designed to measure cerebral hemodynamic changes before and after exercise activity explored the effects of exercise on cognition, with the prefrontal lobe being a key region. In contrast, studies designed to measure cerebral hemodynamics during exercise intervention targeted more locations in the sensorimotor areas (Endo et al., 2013; Auger et al., 2016; Kriel et al., 2016; Monroe et al., 2016; Kenville et al., 2017; Herold et al., 2019; Carius et al., 2020) or other brain areas.

Several exercise processes require a combination of physical activity and cognitive engagement. Hence, it is critical not only to place channels in the motor cortex but also to consider its impact on the prefrontal cortex. Advances in technology have led to an increase in the number of channels for portable devices, thus enabling the measurement of cerebral hemodynamics in multiple ROIs. Given the potential for signal quality issues for measurement during exercise, it is recommended to establish a short-distance channel and instruct participants to minimize head movement during the testing procedure.

### 4.3 Exercise intervention

Among studies designed to measure fluctuations in cerebral hemodynamics before and after exercise interventions, both long-term exercise interventions and short-term exercise interventions were available. Conversely, few long-term interventions were investigated, most likely due to challenges in conducting the assessment over extended periods. Exercise protocols for long-term interventions were flexible, featuring a diverse range of exercise protocols without overlap between studies and a uniform exercise frequency of 2 to 3 sessions per week (Eggenberger et al., 2016; Coetsee and Terblanche, 2017; Lai et al., 2020; Yang et al., 2020). Only one study applied an exercise frequency of 5 times per week (Chen et al., 2017). Similarly, exercise intensity and duration were relatively consistent, with all being of moderate intensity (Eggenberger et al., 2016; Chen et al., 2017; Coetsee and Terblanche, 2017; Lai et al., 2020; Yang et al., 2020) and each session lasting over 30 min (Chen et al., 2017; Lai et al., 2020; Yang et al., 2020).

At present, studies focusing on the effects of long-term exercise interventions on brain functional activity using fNIRS predominantly aim to detect changes in cognitive-task-related brain functional activity before and after exercise interventions but do not involve the detection of brain functional activity during the exercise task. In studies designed to measure changes in cerebral hemodynamics during acute exercise intervention, all exercise types were relatively easy to implement in the laboratory, requiring minimal activity space and offering flexibility, such as cycling and running. These factors may account for the fact that only three exercise programs were used. In the future, it might be possible to further explore changes in cerebral blood flow during other commonly practiced exercises that involve minimal head movement, such as Tai Chi Chuan and yoga.

The length of the single exercise session, on the other hand, did not exceed 30 min (Yanagisawa, 2010; Hyodo, 2012; Endo et al., 2013; Wen et al., 2015b; Lambrick et al., 2016; Coetsee and Terblanche, 2017; Hashimoto et al., 2018; Kujach, 2018; Ji et al., 2019; Xu et al., 2019; Stute et al., 2020; Fujihara et al., 2021; Miyashiro et al., 2021; Zhang et al., 2021), with the majority lasting for only 10 min (Yanagisawa, 2010; Hyodo, 2012; Byun et al., 2014; Wen et al., 2015a,b; Kujach, 2018; Kim et al., 2021). Consequently, changes in cerebral hemodynamics during prolonged exercise sessions remain enigmatic.

To explore hemodynamic changes during exercise, future studies may extend the duration of exercise sessions to 30 min to determine the effect of long exercise session durations on cerebral cortical blood flow.

### 4.4 Effect of exercise on cerebral cortical blood flow changes

In the exploration of task designs, the activation of hemoglobin was frequently discussed, with most studies using oxygenated hemoglobin as an indicator to evaluate brain activation (Yanagisawa, 2010; Wen et al., 2015a,b; Eggenberger et al., 2016; Jiang and Wang, 2016; Chen et al., 2017; Hashimoto et al., 2018; Kujach, 2018; Ji et al., 2019; Xu et al., 2019; Lai et al., 2020; Yang et al., 2020; Fujihara et al., 2021; Kim et al., 2021; Miyashiro et al., 2021; Zhang et al., 2021).

Achieving consistent results with long-term exercise interventions was challenging due to the diversity of the tasks performed. Interestingly, although the adopted tasks were different, most studies identified changes in the left PFC during inhibitory control tasks (Chen et al., 2017; Coetsee and Terblanche, 2017; Yang et al., 2020). In general, higher hemoglobin activity was observed in the cortical region after the cessation of one-time exercise compared with baseline cortical activity, similar to changes in cortical activation during cognitive tasks. Besides, alterations in the prefrontal cortex and motor cortex were observed during one-time exercise sessions.

Considering that fNIRS signals are significantly impacted by physiological artifacts of the system (Caldwell et al., 2016), the influence of exercise on the cerebral cortex is assumed to mainly arise from physiological confounders after exercise. According to the findings of a methodological investigation, fNIRS signals are affected by systemic physiological artifacts for up to approximately 8 min after stopping cycling for 10 min (Byun et al., 2014). Therefore, the results of studies that performed fNIRS tests after exercise (<~8 min) should consider the effect of physiological confounds. However, a reasonable hypothesis suggests that some fNIRS signals observed post-exercise originated from neuronal activity. The entire prefrontal cortex should be impacted by systemic physiological changes if the greater cortical activity observed after exercise is primarily a result of systemic physiological artifacts. The fact that greater cortical activity was observed only in specific regions of the prefrontal cortex rather than the entire prefrontal cortex is significant because it supports the hypothesis that the fNIRS signals were at least partially derived from neuronal activity. The positive neurobehavioural correlation between cognitive ability and cortical activity in various regions of the prefrontal cortex further supports this notion.

The review indicated that exercise interventions alter oxygenated hemoglobin levels in the prefrontal cortex and motor cortex, which are associated with improvements in higher cognitive functions such as inhibitory control and working memory. These findings further support the hypothesis that exercise promotes changes in the prefrontal and motor cortex, which may be key regions for exercise to promote brain health. To deepen our understanding of the fundamental processes by which exercise modifies the brain, future studies should concentrate on alterations in cerebral blood flow during physical exertion.

### 4.5 Limitations

Nevertheless, this review has limitations that merit acknowledgment. To begin, this review only explored studies involving exercise durations over 10 min, but future studies should expand their scope to explore durations of exercise that contribute to brain health. The primary purpose of this review was to conduct subgroup analysis based on different research designs (measuring fNIRS before, during, and after exercise intervention). Future analyses should aim to stratify group populations or exercise types into different subgroups. The systematic search, dual-author screening, eligibility assessment, and quality appraisal were employed to minimize biased selection of studies, whilst dual-author auditors ensured a thorough search. However, this review was not preregistered and not available for inspection by other researchers, and future reviews can be registered with PROSPERO in advance. A meta-analysis was not conducted due to the high degree of heterogeneity across studies and the low number of studies examining each outcome.

## 5 Conclusions and future perspectives

Overall, fNIRS is a promising neuroimaging tool that provides insights into changes in exercise-induced hemodynamics before, during, and after exercise interventions.

Studies using fNIRS to measure cerebral blood flow before and after exercise interventions have predominantly incorporated relatively few long-term interventions and predominantly featured short-term interventions. These studies have mainly focused on the effects of exercise on brain activity during cognitive tasks such as inhibitory control. However, the study equipment and study intervention protocols were less stringent and relatively more flexible. All studies on this topic have focused on changes in oxygenation in the prefrontal area of interest.

Few of the included studies measured hemodynamic changes during exercise, with all of them employing short-term interventions, relatively fixed exercise intervention protocols, minimal head motion, and short exercise durations. Of note, the exercises were easy to execute in the laboratory setting, and the fNIRS systems had a limited number of channels.

Exercise protocols for long-term interventions encompassed a wide range of exercise protocols and an exercise frequency ranging from 2 to 3 sessions per week. The exercise intensity was moderate, and the duration of a single session exceeded 30 min. Exercise types for long-term interventions were those that necessitated minimal space and were flexible, such as cycling and running. The duration of a single exercise session did not exceed 30 min, with most sessions lasting for 10 min. Finally, exercise intensities were maintained at a moderate level.

The results of this review signaled that exercise interventions alter oxygenated hemoglobin levels in the prefrontal cortex and motor cortex, which are associated with improvements in higher cognitive functions such as inhibitory control and working memory. The frontal cortex and motor cortex may be key regions for exercise to promote brain health. Future research could further focus on changes in cerebral blood flow during exercise to better understand the underlying mechanisms by which exercise influences brain function.

Moreover, due to its insensitivity to motion, the fNIRS is ideally suited for research during exercise interventions. It is paramount to thoroughly scrutinize the study design, fNIRS device parameters, and exercise protocols. Cerebral blood flow during exercise intervention represents a future research direction that has the potential both to identify cortical hemodynamic changes and elucidate the relationship between exercise and cognition.

Future studies can combine two study designs that measure blood flow before, during, and after exercise in a more in-depth and comprehensive manner. Additionally, they can utilize multiple indicators, such as functional connectivity, to accurately reflect the effects of exercise on brain functional networks.

## Data availability statement

The original contributions presented in the study are included in the article/Supplementary material, further inquiries can be directed to the corresponding authors.

## Author contributions

Q-QS: Writing – original draft, Writing – review & editing. J-MH: Writing – review & editing. TX: Writing – review & editing. J-YZ: Writing – review & editing. D-LW: Writing – review & editing. YY: Writing – review & editing. RL: Writing – review & editing. Z-LX: Writing – review & editing. H-cY: Supervision, Writing – review & editing, Funding acquisition, Project administration. LC: Supervision, Writing – review & editing.
