# Supplementary material for: Exercise promotes brain health: a systematic review of fNIRS studies
Source: Front Psychol. 2024 Apr 10;15:1327822. doi: 10.3389/fpsyg.2024.1327822 (PMC11042249; doi:10.3389/fpsyg.2024.1327822)
Supplement: Supplementary file 1 [file Table_1.DOCX]

| **First author** | **Year** | Age | **Group or condition**  **comparison** | **fNIRS state and task** | **Duration of fNIRS task** | **Physiological outcome index** | **Behavioural**  **outcome index** | **Instrument type** | **Sampling Frequency** | **Diode** | **Laser** | **Channel** | **Duration**  **(weeks)** | **Sessions/**  **week** | **Session length**  **(min)** | **Exercise type** | **Exercise intensity** | **fNIRS Result reprocessing** | **Task fNIRS data analyse** | **1. ROI**  2. Oxygenation index | **Results** |
| --- | --- | --- | --- | --- | --- | --- | --- | --- | --- | --- | --- | --- | --- | --- | --- | --- | --- | --- | --- | --- | --- |
| **Yanagisawa (22)** | 2010 | 21.5±4.8 | ex condition;  con condition | Task state: Stroop task | 7.5min | NA | NA | ETG-7000 | 100ms | 16 | 16 | 48 | NA | NA | 10 min | cycling | Moderate:  50% of VO_2_ peak | (1)Individual timeline data for the oxy-Hb signal of each channel were preprocessed with a bandpass filter using cut-off frequencies of 0.04 Hz to remove baseline drift and 0.7 Hz to filter out heartbeat pulsations.  (2) From the preprocessed time series data, we obtained channel-wise and subjectwise contrast by calculating the inter-trial mean of differences between the oxy-Hb signals of peak (4–11 s after trial onset) and baseline (0–2 s before trial onset) periods. | We obtained channel-wise and subject-wise contrast by calculating the inter-trial mean of differences between the oxy-Hb signals of peak (4–11 s after trial onset) and baseline (0–2 s before trial onset) periods. The contrasts obtained were subjected to second level, random effects group analysis. | 1.anterior VLPFC; left DLPFC; left FPA; right DLPFC  2.oxy-Hb | **Pre VS Post**  **ex group and con group:** ↑ left DLPFC |
| **Hyodo (23)** | 2012 | 69.3±3.5 | ex condition;  con condition | Task state: Stroop task | 13min | HR | RPE | ETG-7000 | 10Hz | 16 | 16 | 48 | NA | NA | 10 min | cycling | Moderate:  50% of VO_2_ peak | (1) Individual timeline data for the oxy- and deoxy-Hb signal of each channel were preprocessed with a bandpass filter using cutoff frequencies of 0.04 Hz to remove baseline drift and 0.7 Hz to filter out heartbeat pulsations.  (2) From the preprocessed time series data, we obtained channel-wise and subject-wise contrasts by calculating the intertrial mean of differences between the oxy- and deoxy-Hb signals of peak (6 – 8 seconds after trial onset for oxy-Hb and 7–9 seconds after trial onset for deoxy-Hb) and baseline (0 –2 seconds before trial onset) periods. | Averaged the (incongruent - neutral) contrasts for these 2 conditions for each subject before the group analyses, and performed a ROI-wise analysis. | 1.bilateral DLPFC, VLPFC, FPA  2.oxy-Hb; deoxy-Hb | **con group VS. ex group**  **Pre:** ↑ bilateral DLPFC, VLPFC, FPA (oxy-Hb)  ↑ DLPFC (deoxy-Hb)  **Post:** ↑ right FPA (oxy-Hb) |
| **Endo (8)** | 2013 | 23±1 | 20% EX_max_ condition;  40% EX_max_ condition;  60% EX_max_ condition;  con condition | Resting state: Sit 5 min  Task state: Stroop task,  exercise task | exercise task: 15min | MAP; HR | RPE | NIRO 200 | 1Hz | NR | NR | NR | NA | NA | 15 min | cycling | 20, 40, and 60% of EX_max_ | The prefrontal Oxy-Hb and Deoxy-Hb signals were preset to zero before exercise and their average changes over the whole 15-min period of exercise were calculated. | **Stroop task:** With respect to the Stroop 1 test before exercise, the changes in HR and MAP were measured at the end of the Stroop test, and the average changes in the NIRS signals over the whole period of the Stroop test were calculated. These cardiovascular and NIRS signal changes were statistically compared with the baseline values prior to the Stroop test by a paired t test.  **exercise task:** The prefrontal Oxy-Hb and Deoxy-Hb signals were preset to zero before exercise and their average changes over the whole 15-min period of exercise were calculated. | 1.bilateral PFC  2. oxy-Hb; deoxy-Hb | **baseline vs. during Stroop 1 (before exercise)**  **Without exercise:** ↑ bilateral PFC (oxy-Hb)  **20% EX_max,_ 40% EX_max,_ 60% EX_max_:** ↑ bilateral PFC (oxy-Hb)  **baseline vs. during Stroop 2 (after exercise)**  **40% EX_max_:** ↑ bilateral PFC (oxy-Hb)  **Pre VS Post**  **40% EX_max_:** ↑ bilateral PFC (oxy-Hb)  **60% EX_max_:** ↑ bilateral PFC (oxy-Hb)  **Without exercise vs. 40% EX_max_**  **after exercise:** ↑ bilateral PFC (oxy-Hb)  **Without exercise vs. 60% EX_max_**  **after exercise:** ↑ bilateral PFC (oxy-Hb) |
|  |  |  |  | Task state: cycling |  | MAP; HR | RPE | NIRO 200 | 1Hz | NR | NR | NR | NA | NA | 15 min | cycling | 20, 40, and 60% of EX_max_ |  |  | 1.bilateral PFC  2. oxy-Hb; deoxy-Hb | **baseline vs. during cycling task**  **60% EX_max_:** ↑ bilateral PFC (oxy-Hb)  **Without exercise vs. 60% EX_max:_**  ↑ bilateral PFC (oxy-Hb) |
| **Kujach (24)** | 2013 | 21 | HIE condition;  con condition | Task state: Stroop task | 6.5min | HR | RPE;  TDMS | ETG-7000 | 10Hz | 8 | 8 | 48 | NA | NA | 10 min | cycling | 60% of MAP | (1) We preprocessed individual timeline data for the oxy- and deoxy-Hb signal of each channel using the band filter with a cut-off frequency of 0.04 Hz to remove baseline drift and 0.3 Hz to filter out heartbeat pulsations.  (2) We obtained channel-wise and subject-wise contrasts from the preprocessed time-series data by calculating the inter-trial mean of differences between the oxy- and deoxy-Hb signals of peak (4−11 s after the onset of trial) and baseline (0−2 s before the onset of trial) periods. | Specifically, the following contrast, {[(incongruent – neutral) of post-session] – [(incongruent – neutral) of pre-session] in HIE condition} – {[(incongruent – neutral) of post-session] – [(incongruent – neutral) of pre-session] in Con condition}, was calculated for each variable. | 1.bilateral DLPFC, VLPFC, FPA  2. oxy-Hb | **resting control vs. HIE**  **Post:** ↑ left DLPFC  **Pre VS Post**  **HIE:** ↑ left DLPFC  **resting control:** ↑ right VLPFC |
| **Miyashiro (25)** | 2013 | 20-24 | Meditation condition;  ex condition;  con condition | Task state: N-back task | NR | NA | NA | ETG-4000 | 10Hz | 8 | 7 | 22 | NA | NA | 20 min | push-ups | Not reported | No reported. | NR | 1.DLPFC and OFC, Frontopolar prefrontalCortex  2. oxy-Hb | **meditation-control pair and the exercise-control pair pair vs. the meditation-exercise**  **Post:**↑ right and left OFC |
| **Byun (26)** | 2014 | 20.6±1 | ex condition;  con condition | Task state: Stroop task | 6.5min | NA | RPE;  TDMS | ETG-7000 | 10Hz | 16 | 16 | 48 | NA | NA | 10 min | cycling | Light:  30% of VO_2_ peak | (1) Individual timeline data for the oxy- and deoxy-Hb signal of each channel were preprocessed with a band filter using a cut-off frequency of 0.04 Hz to remove baseline drift and 0.3 Hz to filter out heartbeat pul sations.  (2) Channel-wise and subject-wise contrasts were obtained from the preprocessed time series data by calculating the inter-trial mean of differences between the oxy- and deoxy-Hb signals of peak (4–11 s after trial onset) and baseline (0–2 s before trial onset) periods. | Channel-wise and subject-wise contrasts were obtained from the preprocessed time series data by calculating the inter-trial mean of differences between the oxy- and deoxy-Hb signals of peak (4-11 s after trial onset) and baseline (0-2 s before trial onset) periods. The contrasts obtained were subjected to a second level of random effects group analysis. | 1.bilateral DLPFC, VLPFC, FPA  2. oxy-Hb; deoxy-Hb | **con group vs. ex group**  **Post:**↑ left DLPFC (oxy-Hb)  **Post:**↑ left FPA (oxy-Hb) |
| **Wen (27)** | 2015a | 23.6±1.2 | ex condition;  con condition; | Task state: Flanker task | NR | HR | RPE | ETG-4000 | 10Hz | 16 | 16 | 44 | NA | NA | 10 min | cycling | Moderate:  66% of HR_max_ | Preprocess the fNIRS raw data to filter out components with frequencies less than 0.04Hz and greater than 0.7Hz. After preprocessing, the mean oxy-Hb signal under different task levels and experimental conditions was calculated. | The difference between the oxy-Hb signals of inconsistent tasks and consistent tasks | 1.bilateral DLFPC, FPA, VLFPC  2. oxy-Hb | **con group vs. ex group**  **Post:**↑ left PA |
| **Wen (28)** | 2015b | 58.7±7.2 | ex condition;  con condition; | Task state: Flanker task | NR | HR | RPE | ETG-4000 | 10Hz | 16 | 16 | 44 | NA | NA | 10 min | cycling | Moderate:  66% of HR_max_ | (1) Filtering processing: retain the components between 0.04~0.7Hz.  (2) Integration processing: count the oxy-Hb signals at the consistent task and inconsistent task levels in sequence according to the task type. | The difference between the oxy-Hb signals of inconsistent tasks and consistent tasks | 1.bilateral DLFPC, FPA, VLFPC  2. oxy-Hb | **con group vs. ex group**  **Post:**↑ right DLPFC, right FPA |
| **Eggenberger (9)** | 2016 | 74.9±6.9 | video game dance group;  balance and stretching group | Task state: Walking task | 18min | NA | TMT-A; TMT-B; Stroop task; Executive Control task; MoCA; SPPB; FES-I; GDS | Oxiplex TS Tissue Spectrometer | 1Hz | 8 | 2 | NR | 8 weeks | 3 | 30 min | DANCE or BALANCE | Moderate-vigorous | Data from the two sensors on the left and right PFC, respectively, were analyzed separately. Raw data were de-trended and transformed to concentration change values (" µM) by subtracting a 60s moving average as a high-pass filter. After visual inspection of the variation range of the data, motion artifacts in HbO2-values were defined as >2.5 and < −2.5µM and were excluded from further analyses.Artifact cut-off for Hb-values was defined as >1.5 and < −1.5 µM. | Time-triggered averages were calculated for the eight 1-min walk/rest blocks containing preferred walking intervals and the eight blocks with fast walking intervals. | 1.bilateral PFC  2. oxy-Hb | **Pre VS Post**  **Preferred t1-7:** ↓ left PFC, right PFC  **Fast walking:** ↓ left PFC  **Preferred** VS **Fast Walking Speeds t1-7**  **Post:** ↓ right PFC  **Preferred vs. Fast Walking Speeds t10-25**  **post :** ↓ left PFC  **Baseline vs. Experimental**  **Preferred and Fast Walking Speeds t1-7, t10-25, t26-34,** ↓ left PFC, right PFC  **referred and Fast Walking Speeds t35-46:**↑ left PFC, right PFC |
| **Jiang (29)** | 2016 | 20.6 | ex condition; | Task state: Flanker task | NR | NA | NA | NR | NR | NR | NR | NR | NA | NA | 20 min | cycling | Moderate intensity | (1) The NIRS raw data are preprocessed to filter out components with frequencies less than 0.04Hz and greater than 0.7Hz.  (2) After preprocessing, the mean value of oxygenated hemoglobin (oxy-Hb) signal under different experimental conditions in the left and right brain regions was calculated. | NR | 1.bilateral frontal area  2.oxy-Hb | **Pre VS Post**  **20 min of moderate intensity aerobic ex group:** ↑ Bilateral frontal area |
| **Lambrick (38)** | 2016 | 8.8±0.8 | CONT condition;  INT condition | Task state: Stroop task | NR | HR; VO_2_; V_E_; RER; energy expenditure | Eston-Parfitt Scale | PortaLite | 2Hz | 3 | 3 | NR | NA | NA | 30 min | running | Submaximal | (1) NIRS data (O2Hb, HHb, tHb) from CONT and INT were filtered by Gaussian smoothing and exported at a sample rate of 2 Hz.  (2) NIRS data for each time point (pre, 1min_post, 15min_post, 30min_post), which are typically measured in micromol/L, were calculated as a proportion of the resting baseline value, to facilitate interindividual comparisons, and used in the subsequent analyses. | NIRS data for each time point (pre, 1min_post, 15min_post, 30min_post), which are typically measured in micromol/L, were calculated as a proportion of the resting baseline value, to facilitate interindividual comparisons, and used in the subsequent analyses. | 1.the supraorbital ridge of the participant’s dominant side  2. oxy-Hb;  deoxy-Hb;  total Hb | **Pre vs. 1 min_post**  **CONT and INT:** ↑ supraorbital ridge of the dominant side (oxy-Hb), supraorbital ridge of the dominant side (total Hb)  **1 min_post vs. 15 min_post**  **CONT and INT:** ↓ supraorbital ridge of the dominant side (oxy-Hb), supraorbital ridge of the dominant side (Total-Hb)  **Pre vs. 15 min_post**  **CONT and INT:** ↑ supraorbital ridge of the dominant side (oxy-Hb), supraorbital ridge of the dominant side (total Hb)  **Pre vs. 30 min_post**  **CONT and INT:** ↑ supraorbital ridge of the dominant side (oxy-Hb), supraorbital ridge of the dominant side (total Hb)  **1 min_post vs. 30 min_post**  **CONT) and INT:** ↓supraorbital ridge of the dominant side (deoxy-Hb) |
| **Chen (11)** | 2017 | 22.5±2 | BMB group;  con group | Task state: Flanker task | 7.5min | NA | POMS (short version) | ETG-4000 | NR | 6 | 10 | 44 | 8 weeks | 5 | 90 min | BMB | NR | No reported | A 2 (time: before vs. after) × 2 (flanker congruency: congruent vs. incongruent) × 2 (group: intervention vs. control) × 2 (frontal lobe: left vs. right) analysis of variance (ANOVA). | 1.bilateral PFC  2. oxy-Hb; | **Pre VS Post**  **Baduanjin intervention group:** ↑ left PFC |
| **Coetsee (12)** | 2017 | 62.7±5.7 | RT group;  HIIT group;  MCT group;  con group | Task state: Stroop task | NR | walking endurance | NA | NIRO 200NX | 5Hz | 2 | 2 | 2 | 16 weeks | 3 | 30 min | walking | Moderate-vigorous | (1) The mean NIRS values obtained at baseline were subtracted from the mean values attained during the naming and executive Stroop tasks, respectively.  (2) the NIRS changes during the naming task were subtracted from the executive task to give an indication of the Stroop interference effect.  (3) Baseline NIRS values refer to values obtained during the rest period at the given testing session (pre-test and post-test). Pre-test values refer to the measurements obtained during the first testing session. | The mean NIRS values obtained at baseline were subtracted from the mean values attained during the naming and executive Stroop tasks, respectively. Additionally, the NIRS changes during the naming task were subtracted from the executive task to give an indication of the Stroop interference effect. Baseline NIRS values refer to values obtained during the rest period at the given testing session (pre-test and post-test). | 1.bilateral PFC  2. oxy-Hb;  deoxy-Hb;  total Hb | 1. Stroop task  (naming condition)  **Pre VS Post**  **con group:**↑ left PFC (oxy-Hb)  **MCT group:** ↑ left PFC (deoxy-Hb); ↓ left PFC (total Hb)  **con group vs. HIIT group, MCT group, and RT group**:  **post:** ↓ left PFC, significant difference (oxy-Hb) |
|  |  |  |  |  |  |  |  |  |  |  |  |  |  |  |  |  |  |  |  | 1.bilateral PFC  2. oxy-Hb;  deoxy-Hb;  total Hb | 1. Stroop task  (executive condition)  **Pre VS Post**  **RT group:**↓ left PFC (oxy-Hb), left PFC (total Hb);↑ left PFC (deoxy-Hb)  **MCT group:** ↑left PFC (deoxy-Hb); ↓left PFC (total Hb)  **con group vs. HIIT group, and MCT group:**  post: ↓ left PFC, significant difference (oxy-Hb) |
| **Hashimoto (30)** | 2018 | 24.3±3.5 | 40% VO_2_ peak condition;  60% VO_2_ peak condition;  con condition | Task state: Paced Auditory Serial Addition Test | 125s | NA | NA | ETG-4000 | 100ms | 8 | 8 | 24 | NA | NA | 15 min | cycling | 40% and 60% of the peak oxygen | The resting scores were subtracted from the PASAT task scores, and the resulting calculations were averaged using the NIRS. | The resting scores were subtracted from the PASAT task scores, and the resulting calculations were averaged using the NIRS. Next, the difference between the post-exercise and pre-exercise scores was calculated to determine the changes in cerebral blood flow according to each condition. | 1. the frontal and temporal areas  2. oxy-Hb | **40% VO_2_ peak and rest vs. 60% VO_2_ peak**  **Post:** ↑ left PFC |
| **Ji (31)** | 2019 | 65.6±1.32 | CE condition;  PE condition;  CE + PE condition；  RC condition | Task state: Stroop task | 8min | NA | NA | NIRScout | 3.91Hz | 8 | 8 | 20 | NA | NA | 15 min | cycling | 65% of heart rate | (1) The fNIRS signals were bandpass filtered between 0.01 Hz and 0.2 Hz to remove baseline drift, artefacts and physiological noise. The analysis of fNIRS signal was performed by block (i.e., by task, including all trials), without separating out error trials.  (2) the fNIRS data, we calculated the value of the Oxy-Hb change, by subtracting the mean Oxy-Hb of the pre-intervention task period from the mean Oxy-Hb of the post-intervention task period. | Variables of interest were relative changes in concentration of Oxy-Hb and Deoxy-Hb compared to the baseline (1 min at rest before the computerized Stroop task). | 1.bilateral VLPFC, and DLPFC  2. oxy-Hb | Stroop task (naming condition)  **RC condition vs. PE condition**  ↑ right VLPFC |
|  |  |  |  |  |  |  |  |  |  |  |  |  |  |  |  |  |  |  |  | 1.bilateral VLPFC, and DLPFC  2. oxy-Hb | Stroop task(executive conditions)  **RC condition vs. PE condition**  ↑ left DLPFC, right DLPFC  **CE condition vs. PE condition**  ↑ right DLPFC |
| **Xu (32)** | 2019 | 20.7±1.6 | exp condition;  con condition | Task state: Table-setting task | 10min | NA | NA | NIRSport | 7.81Hz | 8 | 8 | 14 | NA | NA | 25 min | cycling | Moderate intensity (65% of VO_2_ peak) | (1) the raw intensity data were converted into optical density. the optical density signal was processed with a wavelet and a bandpass filter .  (2) For this, an IQR of 1.219 corresponding to the recommended α of 0.1 was used for the wavelet filter. The cut-off frequencies of 0.01 and 0.5 Hz were used for the bandpass filter. | The beta-values of Oxy-Hb for each task were compared to the corresponding baseline (15 s prior to table-setting task onset) Oxy-Hb. | 1.left IFG, PMC, rostral IPL, and SPL  2. oxy-Hb | **Activation Pattern Assessed Using Channel-Based Group Analysis**  **exec and obs components in the post-exp condition:** Activated in all channel (left IFG, PMC, SPL, rostral IPL)  **post-exp-obs condition:** Activated in channel 2, 6, 9, 11, and 13 (left IFG, PMC, and SPL)  **post-exp-obs components in the no-exercise conditions (post-ctrl, pre-exp, and pre-ctrl conditions)：**  channel 3, 4, 5, 6, 7, 9, 11, 12, 13, and 14 (left IFG, PMC, rostral IPL, and SPL), According to the spatial map of the 23 subjects in the pre-exp condition and pre-ctrl condition, also found that channels 2, 3, 4, 5, 6, 7, 9, 10, and 13 (IFG, PMC, and rostral IPL), were activated during both action execution and observation.  **Activation Pattern Assessed Using ROI-Based Group Analysis**  **action observation and execution in the post-exp condition:** Activated in left IFG, PMC, rostral IPL, and SPL, except SPL during action execution.  **action observation and execution in all the no-exercise conditions:** the IFG, PMC, rostral IPL, and SPL were significantly activated. Only the activation of the SPL during action execution in the post-ctrl condition and the rostral IPL during action execution in the post-ctrl condition were not significantly activated  **ROI-Based Group Analysis for the Effect of Moderate-Intensity Exercise**  **in action observation, during the postsessions** **(exp/ctrl)**: there were significant differences between the exp and ctrl conditions in all four ROIs.  **in action execution, post-ctrl vs. post-exp:** ↑left IFG |
| **Lai (33)** | 2020 | Boys: 72.3±2.74 months  Girls: 73.2±1.30 months | tennis group;  con group | Task state: N-back task | 5min | NA | Physical fitness | PortaLite | 50Hz | 3 | 1 | 3 | 8 weeks | 2 | 60 min | Tennis | Moderate | (1) A moving average window of 1 s was applied to the O2Hb signals to filter out the noise of the heartbeat frequency.  (2) The fNIRS data were excluded from the analyses when the standard deviations of oxy-Hb during the pre-task period exceeded 0.035.  (3) For the fNIRS signals, the value at the start of the first trial was taken as zero. Changes of [O2Hb] were calculated for approximately 105 s from this point. the 1-back task period for each subject was divided into four time-segments of approximately 105 s each.  (4) The signals were linearly detrended and were filtered using a band-pass filter with a cutoff frequency of 0.01~0.1 Hz. The time series was cubically interpolated at 1 Hz to obtain uniformly spaced time series for spectral and transfer function analysis. | The value at the start of the first trial was taken as zero. Changes of [O_2_Hb] were calculated for approximately 105 s from this point. Therefore, the 1-back task period for each subject was divided into four time-segments of approximately 105s each, the mean values of oxy-Hb for each subject were calculated foifour time segments of 105s. | 1.Left PFC  2. oxy-Hb | **Pre VS Post**  **Tennis intervention group:**↑ left PFC  **Tennis intervention group (**Time segment 1、2、3): ↑ left PFC |
| **Stute (36)** | 2020 | 68.26±3.31  69.7±4.23 | exp group;  con group | Task state: N-back task | 6min45s | HR | RPE | NIRSport | 3.47Hz | 16 | 16 | 38 | NA | NA | 15 min | cycling | Moderate:  50% of VO_2_ peak | (1) the raw optical intensity time series data were converted to changes in optical density (OD) using the hmrIntensity2OD function.  (2) Correction for motion artifacts was performed using wavelet filtering.  (3) applied a band-pass filter (third-order low-pass and fifth-order high-pass Butterworth filter) with cut-off frequencies of 0.01–0.08 Hz to remove physiological noise like cardiac frequency, respiratory frequency, Mayer waves, and very low-frequency oscillations. | All trials related to the same condition and time point were block averaged (time window: -2 to 45 s) using the HomER2 hmrBlockAvg function to recover the mean hemodynamic response. | 1.bilateral DLPFC and VLPFC, IPL and SPL  2. HBdiff | **con group vs. exp group**  ↓ both regions (frontal and parietal) and hemispheres (left and right) at almost all time points |
| **Yang (13)** | 2020 | TCC Group: 66.31±4.25  con Group: 65.92±3.48 | TCC group;  con group | Task state: Flanker task | 5min | NA | NA | ETG-4000 | NR | 16 | 14 | 44 | 8 weeks | 3 | 45 min | TCC | Moderate | (1) The data from the fNIRS were pre-processed by the xTopo software. The components with frequencies of less than 0.04 Hertz and more than 0.50 Hertz were filtered out.  (2) Baseline correction was performed using a linear fitting function. To this end, a linear fit was performed in the 10 s baseline before all of the active task segments and during the post-task baseline. The post-task baseline was determined as the average over the last 10 s of the resting period between active blocks.  (3) The mean values of the oxy-Hb signals of the four ROIs were calculated by averaging the same-condition blocks for each participant under each channel and then averaging the channels of the ROIs. | A linear fit was performed in the 10 s baseline before all of the active task segments and during the post-task baseline. The post-task baseline was determined as the average over the last 10 s of the resting period between active blocks. The mean values of the oxy-Hb signals of the four ROIs were calculated by averaging the same-condition blocks for each participant under each channel and then averaging the channels of the ROIs. | 1. Frontal_Sup_L, Frontal_Inf_L, Frontal_Sup_R, rontal_Inf_R.  2. oxy-Hb | **Pre VS Post**  **TCC group:**↑ Frontal_Sup_L， Frontal_Inf_L |
| **Fujihara (5)** | 2021 | Young adults: 22.68±1.38  Old adults: 68.72±5.26 | young adult group;  older adult group | Task state: reverse Stroop task | 5min | HRR | RPE | OEG-16 | NR | 6 | 6 | 16 | NA | NA | 15 min | running | Moderate:  HRR of 50% | (1) To increase the ratio of signals, each record was converted to a z-score and traces were compared between participants and channels.  (2) The z-scores were calculated as the differences between the mean oxy-Hb levels at rest and those at trial during the incongruent task divided by the standard deviation of the values at rest. Each signal used the mean and standard deviation of the oxy-Hb levels in the last 6 s of rest and the averaged mean oxy-Hb levels of the last 20 s of each task set. | The z-scores were calculated as the differences between the mean oxy-Hb levels at rest and those at trial during the incongruent task divided by the standard deviation of the values at rest. Each signal used the mean and standard deviation of the oxy-Hb levels in the last 6 s of rest and the averaged mean oxy-Hb levels of the last 20 s of each task set. | 1.the left, middle, and right PFC  2. oxy-Hb | **young adult group vs. older adult group**  **Post:**↑ M-PFC |
| **Kim (6)** | 2021 | 22.5±2.2 | Moderate intensity condition;  High intensity condition | Task state: 2-back task | 3min | NA | NA | NIRSIT | 8.138Hz | 24 | 32 | 48 | NA | NA | 10 min | running | Moderate-vigorous:  65% of VO_2_ peak-vigorous 80% of VO_2max_ | (1) the detected light signals in each wavelength were filtered by a band-pass filter (0.00–0.1 Hz) to reduce noise due to environmental noise-related light and body movements.  (2) In addition, the channels with low quality information (signal-to-noise ratio < 30 dB) were removed from the hemodynamic analysis set to prevent misinterpretation.  (3) The multiple trial results were block-averaged individually, and grand-averaging was performed. | The difference in accHbO_2_ (ΔaccHbO_2_) was calculated as the 2-back NIRS value minus the resting NIRS value. | 1.bilateral DLPFC, OFC  2. oxy-Hb | **Pre VS Post**  **Moderate Intensity:** ↓ right DLPFC  **High Intensity:** ↑ left DLPFC |
| **Zhang (7)** | 2021 | ex group: 22.10±1.85  con group: 21.30±2.54 | ex group;  con group | Task state: Implicit cognitive reappraisal task | 96-102s | NA | NA | OMM3000/8 | 33Hz | 9 | 9 | 27 | NA | NA | 30 min | cycling | Moderate:  60%–69% of HR_max_ | (1) the raw signal of oxy-hemoglobin was filtered to minimize noise, global trends, and biological signals, such as heart rate and respiration.  (2) Specifically, a high-pass filter, the Wavelet-MDL de-trending algorithm, was used to remove the global trend, and a low-pass filter, the canonical hemodynamic response function (HRF), was used for temporal smoothing. | NR | 1.NR  2. oxy-Hb | **Con group vs. ex group**  ↓ channels 1, 25  **baseline vs. Implicit cognitive reappraisal task**  ↓ channels 3 and 8  ↑ channels 11, 16, 21, 23, and 27  **Pre VS Post**  **ex group:** ↑ channels 7 and 13 |
| **Kurz (39)** | 2012 | 23.7±1.4 | walking forwards condition;  walking backwards condition | Task state: walking | 20min | NA | Stride time interval | ETG-4000 | 10Hz | 8 | 8 | 24 | NA | NA | 20 min | walking | walking at 0.45 m/s | (1) The measured oxyHb and deoxyHb hemodynamic waveforms were filtered using a 0.01 Hz high pass filter and a 5.0 s moving average.  (2) To increase the signal-to-noise ratio and exclude extraneous biological noise (e.g., respiratory and cardio-artifacts), principal component analyses were conducted on the respective waveforms taken from all channels.  (3) Components with 0.25 or lower correlation with the reference waveform, which corresponded to the expected hemodynamic function, were filtered from the data while those with correlations above 0.25 were passed and included in the final reconstruction of the individual channel time series. | The reference waveform was a trapezoidal function with an upward slope that started at the onset of walking and had a 5 second time to peak, a 25 second peak duration, and a 5 second downward slope. These data were then used to create average oxyHb and deoxyHb trial waveforms for each channel while the participant walked either forward or backward. Changes in the amount of activation in the medial sensorimotor cortices were evaluated relative to their condition-specific baselines, which were determined from the 2.5 s of fNIRS data that immediately preceded the onset of walking. | 1.SMA, SPL, precentral gyrus (PCG), postcentral gyrus (POCG)  2. oxy-Hb; deoxy-Hb | **forwards vs. backwards walking**  ↑SMA, PCG, SPL (oxy-Hb), ↓SMA (deoxy-Hb) |
|  |  |  |  |  |  |  |  |  |  |  |  |  |  |  |  |  |  |  |  |  |  |
| **Auger (40)** | 2016 | 23.1±2.4 | 40% POP condition;  80% POP condition  Rest condition; | Task state: cycling | 2 min warm up+10 min block duration | PPO | NA | custom built | NR | 4 | 4 | NR | NA | NA | 10 min | cycling | a constant power representing 40% and 80% of their individual PPO | Prior to data analysis, datasets acquired for all exercise conditions were inspected for motion artifacts. This systematic review was performed for all source-detector distances and laser sources, i.e. for 16 datasets per condition. Data from subject 2, 11 and 13 were excluded for poor quality data due to an inadequate contact between the optical probe and the subject’s forehead or head movement while cycling. | NR | 1.PFC  2. oxy-Hb; deoxy-Hb; total-Hb | **rest vs. 80%PPO**  ↑ PFC (deoxy-Hb) |
| **Kriel (37)** | 2016 | 23±3 | HIITPASS condition;  HIITACT condition;  REC condition | Task state: cycling | 18min | VO_2_; HR; HHb; power output | NA | PortaLite | 10Hz | NR | NR | NR | NA | NA | 18 min | cycling | vigorous | smoothed using a 10 point moving average before being averaged to 1 s periods.Due to the HHb data being a measure of change from an arbitrarily assigned baseline zero value, the NIRS data are expressed as units of change (μMol) from the mean value of the 30 s of baseline data preceding the start of exercise. | The NIRS data are expressed as units of change (μMol) from the mean value of the 30 s of baseline data preceding the start of exercise (Δ[HHb]). | 1.PFC  2. Δdeoxy-Hb | **HIITPASS** **and HIITACT and REC**  For the mean Δdeoxy-Hb for each bout, differences were found between conditions for Bout 2, Bout 3 and Bout 4, however no significant differences in PFC were found between the two HIIT conditions. For the mean Δdeoxy-Hb within conditions, there were significant increases across bouts, with values increasing over time in the HIITPASS and HIITACT conditions. |
| **Monroe (41)** | 2016 | 21.3±2.4 | SIC condition  CRC condition | Task state: cycling | 5min warm up + 18 min cycling exercise | HR; oxygen uptake; peak power | POMS-B; RPE | OxyMon MKIII | 1000Hz | 2 | 2 | 4 | NA | NA | 18 min | cycling | In the SIC of each 30-s sprint was summed across the four sprints to calculate total work performed (Work SIC) to match the CRC at 70 rpm | (1) Mean baseline oxygenation status (e.g., HbO2 and HHb) was computed from the middle 3 min of resting baseline.  (2) The peak and mean oxygenation status during each sprint and the minimum and maximum oxygenation during recovery were computed from the entire sprint and recovery periods and were baseline-corrected. | Mean baseline oxygenation status (e.g., HbO_2_ and HHb) was computed from the middle 3 min of resting baseline. The peak and mean oxygenation status during each sprint and the minimum and maximum oxygenation during recovery were computed from the entire sprint and recovery periods and were baseline-corrected. | 1.DLPFC  2. Oxy-Hb; Deoxy-Hb | **baseline vs. exercise**  ↑DLPFC(Oxy-Hb)  **CRC vs. SIC**  ↑DLPFC(Oxy-Hb)  **During SIC:**  ↑DLPFC (Deoxy-Hb) |
| **Kenville (34)** | 2017 | 25.7±2.2 | BS with 0% 1RM (L0%) condition;  BS with 20% 1RM (L20%) condition;  BS with 40% 1RM (L40%) condition;  BL condition | Task state: BS | 25min | NA | NA | NIRSport | 7.81Hz | 8 | 8 | NR | NA | NA | 25 min | barbell load | 0% 1RM, 20% 1RM and 40% 1RM for a BS | fNIRS channels were removed when respective channels exceeded a variation coefficient of 15%. Subsequently data was subjected to a baseline correction (10 s before onset) and then filtered (band pass filter: low cutoff frequency = 0.01 Hz high cutoff frequency = 0.2 Hz) to attenuate high-frequency noise and cardiovascular artifacts. | Conditions (BS during 0% 1RM (no load, L0%), 20% 1RM (L20%) and 40% 1RM (L40%) were modeled using a ‘‘boxcar’’ function folded with the canonic hemodynamic response function. Data were then transformed into a General Linear Model (GLM) estimation to obtain beta values, the conditions and the timely progression of the distinct chromophores HbO_2_ and HHb. | 1. M1, PMC, SMA, IPL, SPL and auditory, frontal and visual areas  2. oxy-Hb; deoxy-Hb | **Alterations in the Haemodynamic Response within Sensorimotor Areas:**  **L0% vs. L20%**  ↑ bilateral M1, right PMC, SMA, left IPL, right SPL (oxy-Hb)  **L0% vs. L40%**  ↑ significant alterations in the haemodynamic response in all channels, except channels 12, 18 and 22 (oxy-Hb)  ↑ left SSC and IPL (deoxy-Hb)  **L20% vs. L40%**  ↑ significant alterations in the haemodynamic response in all channels, except channels 4, 9, 12 and 18 (oxy-Hb)  **BL vs. BS during L0%**  ↑ bilateral M1, SSC, SMA, SPL, left IPL and right PMC (oxy-Hb)  **BL vs. BS during L20% and L40%**  ↑ bilateral M1, SSC, SMA, SPL, left IPL and right PMC (oxy-Hb) |
| **Herold (35)** | 2019 | 25.00±3.00 | overground condition;  treadmill walking condition | Task state: walking | 880s | HR; LF/HF ratio | Walking speed | NIRSport | 7.81Hz | 8 | 8 | 14 | NA | NA | approximately 14.7 min | walking | NR | (1) Raw data for all channels were visually inspected, spike artifacts were removed, and faulty channels were removed from subsequent analyses.  (2) All channels were band-pass filtered, with a low-cutoff frequency of 0.01 Hz and a high-cutoff frequency of 0.1 Hz to remove baseline drift and physiological noise, respectively. | To account for systemic physiological changes, calculated delta (Δ) values for oxy-Hb, deoxy-Hb (for each channel) and mean heart rates by subtracting the mean values obtained during treadmill walking from the mean values obtained during overground walking. | 1.bilateral PFC, PMC, SMA  2. oxy-Hb; deoxy-Hb | **During walking**  ↑left PFC, right PFC, left PMC (oxy-Hb)  **overground vs. treadmill walking**  ↑ left PFC, right PFC, left PMC, right PMC, bilateral SMA (oxy-Hb) |
| **Carius (10)** | 2020 | 24.61±0.47 | DH_slow_ condition;  NDH_slow_ condition;  AH_slow_ condition;  DH_fast_ condition;  NDH_fast_ condition;  AH_fast_ condition | Task state: basketball dribbling | 300s warm up + 756s dribbling | HR | VAS | NIRSport | 7.81Hz | 8 | 7 | 22 | NA | NA | 12.6 min | basketball slalom dribbling | slow walking pace (0.87 ms-1) and fast walking pace (1.75 ms-1) | (1) The fNIRS signal pre-processing steps to reduce the influence of motion artifacts and physiological noise were applied as in Carius et al.  (2) Raw intensity signals were converted to changes in optical density.  (3) Correction for motion artifacts was performed using a hybrid method that takes advantage of different correction algorithms, so-called Spline interpolation with Savitzky-Golay (SG) filtering.  (4) Subsequent to motion-artifact correction, data was band-pass filtered to attenuate low frequency drift, Mayer wave, breathing rate and heart rate components using 0.01 Hz as high and 0.09 Hz as low pass cutoff frequencies. | Single trials of BSDT were baseline corrected (5 seconds until stimulus onset) and time courses of ΔHb and ΔHHb for each channel were block-averaged, analyzed the height of amplitude (baseline-corrected average of the temporal window from 5 to 21 seconds with regard to stimulus onset for ball dribbling. | 1.bilateral PMC-SMA, SAC, M1, IPC  2. oxy-Hb; deoxy-Hb | **AH_slow_ vs.** **DH_slow_**  ↓ IL-M1 (deoxy-Hb)  **slow vs. fast**  **dominant right hand (DH):** ↓ contralateral PMC-SMA (deoxy-Hb) |

HIE = high-intensity intermittent exercise; con = control; EX = exercise; HIIT = high-intensity interval training; RT = resistance training; MCT = moderate continuous training; EX_max_ = maximum voluntary exercise; INT = intermittent bout of exercise; CE = cognitive exercise; PE = physical exercise; CE + PE = cognitive + physical exercise; RC = reading control; exp = experimental; CONT = continuous bout of exercise; NR = Not Reported; NA = Not Applicable.
